# Tick-borne encephalitis virus inhibits rRNA synthesis and host protein production in human cells of neural origin

**DOI:** 10.1371/journal.pntd.0007745

**Published:** 2019-09-27

**Authors:** Martin Selinger, Hana Tykalová, Ján Štěrba, Pavlína Věchtová, Zuzana Vavrušková, Jaroslava Lieskovská, Alain Kohl, Esther Schnettler, Libor Grubhoffer

**Affiliations:** 1 Institute of Parasitology, Biology Centre of the Czech Academy of Sciences, Branišovská 31, České Budějovice, Czech Republic; 2 Faculty of Science, University of South Bohemia in České Budějovice, Branišovská, České Budějovice, Czech Republic; 3 MRC-University of Glasgow Centre for Virus Research, Glasgow, Scotland, United Kingdom; 4 Bernhard-Nocht-Institute for Tropical Medicine, Bernhard-Nocht-Str. 74, Hamburg, Germany; 5 German Centre for Infection Research (DZIF), partner site Hamburg-Luebeck-Borstel-Riems, Hamburg, Germany; University of Texas Medical Branch, UNITED STATES

## Abstract

Tick-borne encephalitis virus (TBEV), a member of the genus *Flavivirus* (*Flaviviridae*), is a causative agent of a severe neuroinfection. Recently, several flaviviruses have been shown to interact with host protein synthesis. In order to determine whether TBEV interacts with this host process in its natural target cells, we analysed *de novo* protein synthesis in a human cell line derived from cerebellar medulloblastoma (DAOY HTB-186). We observed a significant decrease in the rate of host protein synthesis, including the housekeeping genes HPRT1 and GAPDH and the known interferon-stimulated gene viperin. In addition, TBEV infection resulted in a specific decrease of RNA polymerase I (POLR1) transcripts, 18S and 28S rRNAs and their precursor, 45-47S pre-rRNA, but had no effect on the POLR3 transcribed 5S rRNA levels. To our knowledge, this is the first report of flavivirus-induced decrease of specifically POLR1 rRNA transcripts accompanied by host translational shut-off.

## Author summary

Tick-borne encephalitis virus (TBEV) is a causative agent of a severe human neuroinfection that threatens Europe and Asia. Little is known about the interaction of this neurotropic virus with neural cells, even though this may be important to better understand why or how TBEV can cause high pathogenicity in humans, especially following neural cell infection. Here, we showed that TBEV induced host translational shut-off in cells of neural origin. In addition, TBEV interfered also with the expression of host ribosomal RNAs. Interestingly, the transcriptional shut-off was documented for rRNA species transcribed by RNA polymerase I (18S rRNA, 28S rRNA and their precursor 45-47S pre-rRNA), but not for RNA polymerase III rRNA transcripts (5S rRNA). Artificial inhibition of host translation using cycloheximide resulted in the decrease of all rRNA species. Based on these data, TBEV seems to specifically target transcription of RNA polymerase I. These new findings further increase our understanding of TBEV interactions with a key target cell type.

## Introduction

The *Flaviviridae* family contains arthropod-borne viruses including medically important pathogens with worldwide distribution and impact, such as dengue virus (DENV), yellow fever virus (YFV), West Nile virus (WNV), Japanese encephalitis virus (JEV), Zika virus (ZIKV), and tick-borne encephalitis virus (TBEV) [1].

TBEV causes a severe neuroinfection known as tick-borne encephalitis, which affects thousands of people across Eurasia annually [2, 3]. In recent years, an increase in TBEV infection rates in affected countries and in its geographical distribution has been observed, involving previously unaffected areas such as Switzerland and northern Germany [4–6]. Although the disease is not always fatal (mortality rate of 1–2%), a high percentage of patients (35–58%) suffer from permanent sequelae, such as cognitive or neuropsychiatric afflictions, balance disorders, headaches, dysphasia, hearing defects, and spinal paralysis after overcoming the main symptoms [2, 7]. Specific antiviral therapy for tick-borne encephalitis does not exist. Neurons are the primary target for TBEV infection in mice and humans, and according to *post mortem* studies of TBEV-infected patients, the cerebellum is one of the main foci affected [8–10]. Understanding the interaction between TBEV and human neural cells is essential as it could lead to possible new treatment targets and a better understanding of why TBEV infection can result in severe neurological symptoms. Like all flaviviruses, TBEV is an enveloped virus with a single-stranded RNA (ssRNA) genome of positive polarity (approx. 11 kb) with a 7-methylguanosine cap at the 5´end. The coding segment is flanked on both ends by untranslated regions (UTR). Viral proteins are encoded in a single open reading frame that is translated in one poly-protein which is then proteolytically processed into three structural (C, prM, E) and seven non-structural proteins (NS1, NS2A, NS2B, NS3, NS4A, NS4B, NS5) [11–13]. While the structural proteins are the main building units of the viral particle, the non-structural proteins are crucial in the TBEV life cycle. They are essential components of viral replication within the host endoplasmic reticulum or Golgi apparatus-derived membrane compartments and the virion assembly processes and are involved in immune response evasion/counteractions [14–16].

Virus replication is reliant on the host protein synthesis apparatus and manipulates it in favour of viral requirements. There are various strategies viruses use to accomplish this goal and generally aim at three levels: host translational shut-off, processing of host mRNA, and host transcriptional shut-off [17, 18]. Translation of eukaryotic and viral proteins is often controlled at the rate-limiting step of initiation and viruses such as Bunyamwera virus, influenza A virus or poliovirus were shown to target initiation factors [19–23]. More specifically for flaviviruses, a recent study [24] documented repression of the host translation initiation step during DENV infection and general translational repression was also recorded for WNV and ZIKV. While inducing host translational shut-off, viral proteins are still synthesised thanks to alternative translation initiation strategies, such as cap-independent translation [20, 25–27].

Transcription in eukaryotic organisms is carried out by three RNA polymerases: RNA polymerase I, II, and III. Each of the RNA polymerase complexes is responsible for the transcription of different genes. RNA polymerase I (POLR1) yields a single transcription unit 45-47S pre-rRNA, which undergoes a complex maturation process that generates 5.8S, 18S, and 28S rRNA [28, 29]. RNA polymerase III (POLR3) produces 5S rRNA, tRNAs, and specific small RNAs [29]. RNA polymerase II (POLR2) transcribes protein-coding genes and certain small RNAs [30]. Out of all the transcripts synthesised in the eukaryotic cell, ribosomal RNA is the most abundant and a key component of ribosomes. Virus-induced interference with transcription and subsequent processing of host rRNA has been described for influenza A virus [31], herpes simplex virus type I [32], human papillomavirus type 8 [33], human cytomegalovirus [34], and human immunodeficiency virus (HIV) [35]. However, this was not described for flaviviruses.

Given the indications for flaviviruses affecting host translation [24], we aimed at exploring this topic further in TBEV infection of naturally permissive host cells of neural origin, that represent a key cell type responsible for tick-borne encephalitis manifestation. We found that TBEV triggered host translational shut-off that involved lowered expression of GAPDH and HPRT1 housekeeping genes as well as the interferon-induced protein viperin. TBEV further specifically impaired the production of POLR1-transcribed rRNAs. Therefore, we postulate that TBEV specifically targets POLR1-mediated transcription of rRNA and rate of host translation thus promoting virus replication.

## Methods

### Cell lines

The human medulloblastoma (DAOY HTB-186; ATCC), human lung adenocarcinoma (A549; a gift from R. Randall, University of St. Andrews, UK), and Vero (green monkey kidney; Biology Centre, CAS, CZ) cell lines were grown in low glucose DMEM medium supplemented with 10% foetal bovine serum (FBS), 1% antibiotics-antimycotics (amphotericin B 0.25 μg/ml, penicillin G 100 units/ml, streptomycin 100 μg/ml), and 1% L-alanyl-L-glutamine. DAOY HTB-186 cell line is derived from desmoplastic cerebellar medulloblastoma of a 4-year-old Caucasian male [36]. A549s are derived from a lung cancerous tissue (alveolar basal epithelial cells) of a 58-year-old Caucasian male [37]. Vero cells are derived from kidney epithelial cells from African green monkey (*Cercopithecus aethiops*). PS cells (porcine kidney stable) were grown in L15 medium with 3% new-born calf serum (NCS), 1% antibiotics-antimycotics, and 1% L-alanyl-L-glutamine [38]. The human osteosarcoma cell line MG-63 (Sigma-Aldrich) was grown in RPMI 1640 medium supplemented with 10% FBS, 1% antibiotics-antimycotics, 1% L-alanyl-L-glutamine, and 50 nM β-mercaptoethanol. These were explanted from a 14-year-old Caucasian male [39].

For metabolic labelling experiments, all cell lines were grown in RPMI 1640 medium supplemented with 10% FBS, 1% antibiotics-antimycotics, 1% L-alanyl-L-glutamine, and 50 nM β-mercaptoethanol. All cell lines were grown at 37°C and 5% CO_2_; with the exception of PS cells (37°C without additional CO_2_).

### Transfection and plasmids

PolyJet *In Vitro* Transfection Reagent (SignaGen; #SL100688) was used for transfection. The procedure was carried out according to the manufacturer’s protocol. For GFP and *Renilla* luciferase expression, the mammalian expression vectors phMGFP (Promega) and pRL-CMV (Promega) were used, respectively. The wt viperin mammalian expression vector was a kind gift from Lisa F.P. Ng (Singapore Immunology Network, Agency for Science, Technology and Research (A* STAR), Singapore), in which the viperin gene with C-terminal c-myc tag is transcribed under the control of the CMV promoter [40].

### Viruses and infection

Two representatives of the West-European TBEV subtype with different degrees of virulence were used–medium (Neudoerfl) and severe (Hypr). Both strains differ in their coding sequences by only 12 nonconservative amino acid substitutions [41], and in the length and structure of the 3´UTR [42]. When mice were infected peripherally, the Hypr strain exhibited pronounced neuroinvasiveness and caused shorter survival than strain Neudoerfl [41]. The low passage TBEV strain, Neudoerfl (fourth passage in suckling mice brains; GenBank accession no. TEU27495), was provided by Prof. F.X. Heinz (Medical University of Vienna, Austria) [43]. The low passage TBEV strain, Hypr (fourth passage in suckling mice brains; GenBank accession no. TEU39292), is available at the Institute of Parasitology, Biology Centre of CAS, České Budějovice, Czech Republic [44]. Viruses were handled under biosafety level 3 conditions.

TBEV was added to the cells one day post seeding. Cells were then incubated for 2 hours, washed with PBS, and finally fresh pre-warmed medium was added. Brain suspension from uninfected suckling mice was used as a negative control.

### Virus titration

Viral titres were determined by plaque assay as described [45], with minor modifications. Briefly, PS cell monolayers (9x10^4^ cells per well) were grown in 24-well plates and incubated with 10x serial dilutions of infectious samples for 4 hours at 37°C. The samples were then covered by 1:1 (v/v) overlay mixture (carboxymethyl cellulose and 2x L15 medium including 6% NCS, 2% antibiotics-antimycotics, and 2% L-glutamine). After five days, medium with overlay was removed, cells washed with physiological solution, subsequently fixed and stained (0.1% naphthalene black in 6% acetic acid solution) for 45 minutes. Virus-produced plaques were counted, and titres are stated as PFU/ml.

### Antibodies and reagents

The following primary antibodies were used: anti-TBEV C polyclonal antibody (produced in-house), anti-TBEV NS3 polyclonal antibody (a kind gift from Dr. M. Bloom, NIAID, USA), anti-HPRT1 Polyclonal Antibody (Thermo Fisher Scientific; #PA5-22281), anti-GAPDH Antibody [EPR16891] (Abcam; #ab181602), Monoclonal Antibody to Mouse Viperin (Hycult Biotech; #HM1016), anti-NPM1 Monoclonal Antibody FC-61991 (Thermo Fisher Scientific; #MA1-1560), and anti-POLR1A Antibody (Abcam; #ab222065). The following secondary/tertiary antibodies were used: HRP Goat Anti-Guinea Pig (Novex; #A18769), HRP Rabbit Anti-Chicken IgY (H+L) Secondary Antibody (Thermo Fisher Scientific; #A16130), HRP Horse Anti-Mouse IgG Antibody (VectorLabs; #PI-2000), HRP Goat Anti-Rabbit IgG Antibody (VectorLabs; #PI-1000), Biotinylated Anti-Streptavidin Antibody (VectorLabs; #BA-0500), AP-conjugated Streptavidin (VectorLabs; #SA-5100), Streptavidin-DyLight 549 (VectorLabs; Cat#SA-5549), Goat Anti-Rabbit IgG-DyLight 594 (Abcam; #ab96897), Goat Anti-Guinea Pig DyLight 594 (Abcam; #ab150188), and Goat Anti-Chicken IgY H&L-DyLight 488 (Abcam; #ab96947).

L-azidohomoalanine (Click Chemistry Tools; #1066–25) and 5-ethynyl-uridine (Click Chemistry Tools; #1261–25) were used for metabolic labelling of nascent proteins or RNA, respectively. Biotin-PEG4-Alkyne (Click Chemistry Tools; #TA105-25) and Biotin Picolyl Azide (Click Chemistry Tools; #1167–25) were used for subsequent detection of incorporated L-azidohomoalanine or 5-ethynyl-uridine, respectively. Cycloheximide was purchased from Sigma-Aldrich (#01810-1G).

### Flow cytometry analysis

DAOY cells were seeded one day prior to infection in the 12-well plate at a density of 2.5×10^5^ cells/well. At the indicated time intervals post-TBEV infection, cells were washed with PBS, trypsinized, and fixed by 4% paraformaldehyde in PBS (Roth). After permeabilization (0.1% Triton X-100), cells were stained using guinea pig anti-TBEV C antibodies (1:1500 dilution) and anti-guinea pig DyLight 594 (1:500 dilution) secondary antibodies. Flow cytometry was performed on a FACS Canto II cytometer and data analysed using FACS DIVA software v. 5.0 (BD Biosciences).

### RNA isolation

Total cellular RNA was isolated using Trizol-based RNA Blue reagent (Top-Bio; #R013) according to the manufacturer’s instructions. RNA pellets were dissolved in DEPC-treated water and directly used for either real-time PCR or analysis on an RNA denaturing gel.

### rRNA quantification

The quantity and integrity of rRNA in total RNA samples were analysed on a 2100 Bioanalyzer using Agilent RNA 6000 Nano kit (Agilent Technologies; #5067–1511). The concentration of each sample was determined spectrophotometrically prior the Bioanalyzer measurement and samples were diluted according to the cell number ratio (resulting concentrations were between 10–20 ng/μl). 1 μl of the diluted RNA samples was loaded on the Bioanalyzer chip and the electrophoretic assay was performed according to the manufacturer’s instructions. All samples were analysed in technical triplicates. 1.2% agarose MOPS-buffered denaturing gel (with 6.7% formaldehyde) was used for fractionation of isolated total RNA. RNA was visualised by addition of the GelRed dye (Biotium) into the gel. The signal was subsequently quantified using Fiji software.

### Sample standardisation

**Fig 1:**
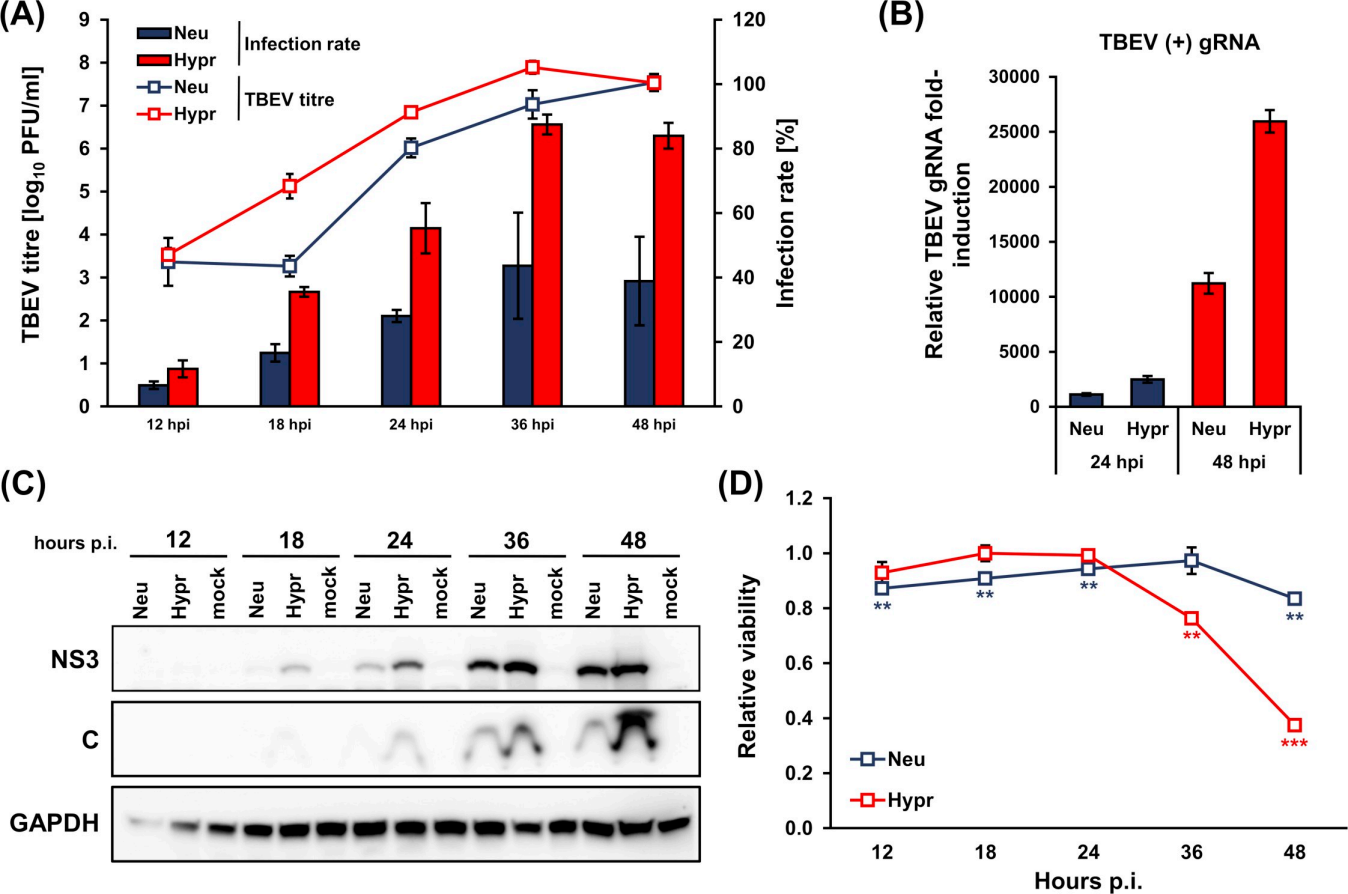
Characterization of TBEV Hypr and Neudoerfl infection kinetics in DAOY cells. DAOY cells were infected with TBEV Neudoerfl or Hypr strain (MOI 5). (A) Viral titres (indicated in trend lines) determined by plaque assay on PS cells and infection rate (indicated in bars) determined by flow-cytometric detection of TBEV C-stained cells were analysed at 12, 18, 24, 36, 48 hours p.i. Graphical summary of three independent experiments is shown with values expressed as mean with SEM. (B) Total RNA was isolated at 24 and 48 hours p.i. and relative qPCR quantification of TBEV gRNA using Δ-c_t_ method with normalisation to cell number was performed. Data are summary of three independent experiments and values in graphs are expressed as mean with SEM. (C) Levels of TBEV NS3 and C proteins in infected DAOY cells were determined by immunoblotting at 12, 18, 24, 36, 48 hours p.i. Data are summary of three independent experiments. (D) Viability of infected cells was determined using alamarBlue reagent, at the indicated time intervals p.i. Data are summary of three independent experiments and values are expressed as mean with SEM, normalised to mock infected cells; significant difference from control was calculated by unpaired Student’s t-test (** P<0.01; *** P<0.001).

We observed that the viability of TBEV Hypr-infected cells was negatively affected at 36 and 48 hours p.i. (Fig 1D). Therefore, in order to diminish the effect of this phenomenon on our data, we decided to standardise in our experiments to cell counts.

Normalisation to cell numbers was performed for real-time PCR, western blotting, northern blotting, and metabolic labelling analyses. For this, we established a viability-based method using alamarBlue reagent (Thermo Fisher Scientific; #DAL1025). Our data demonstrate that viability measurement is directly proportional to the cell number, and therefore this method is fully suitable for normalisation to the cell number (S1 Fig). The procedure was performed according to the manufacturer’s instructions. Briefly, cells were washed with PBS and fresh pre-warmed growth medium with diluted alamarBlue reagent was added (1:10 dilution ratio; v/v). Cells were incubated for 2–2.5 hours and fluorescence of the reduced product was measured on a BioTek plate reader (λ_ex_ = 550 nm; λ_em_ = 590 nm). Growth medium with alamarBlue without cells was used as a blank. All samples were analysed in technical triplicates. Average fluorescence values for TBEV-treated sample were normalized to the respective mock control cells. The viability factor (f) was subsequently used as a normalisation factor for the calculation of RNA/protein input based on the pre-set mock control input.

$$f=\frac{{viability}_{sample}[a.u.]}{{viability}_{control}[a.u.]}\;V_{sample}[\mu l]=\frac{V_{control}[\mu l]}{f}$$

### Real-time qPCR

For real-time qPCR analyses, the KAPA SYBR FAST Universal One-Step qRT-PCR Kit was used according to the manufacturer’s protocol. Data obtained were processed via relative quantification using the delta c_t_ (Δ-c_t_) method; the amount of RNA was adjusted to the cell number instead of the c_t_ values of the housekeeping reference gene. All samples were treated with dsDNase and subsequently 5× diluted in RNAse-free water before the real-time PCR analysis. All samples were analysed in technical triplicates. List of primers used can be found in S1 Table.

### Western blotting

Cells were washed with PBS and RIPA buffer (25 mM Tris-HCl pH 7.6, 150 mM NaCl, 1% NP-40, 0.1% SDS, 1% sodium deoxycholate) with protease inhibitors (Thermo Fisher Scientific; #78430) was added. Cell lysis was performed for 15 minutes on ice while gently shaking. Sonicated and cleared protein lysates in RIPA buffer were separated on 12% denaturing polyacrylamide gels and blotted onto PVDF membranes. The quantity of proteins was normalised to the cell number. Membranes were blocked (5% skimmed milk in PBS-T) and incubated with primary, secondary, and alternatively also tertiary antibodies; between each staining step, membranes were washed three times in PBS-T. Primary antibodies used were guinea pig anti-C (produced in-house; 1:1500), chicken anti-NS3 (M. Bloom laboratory; 1:5000), rabbit anti-GAPDH (Abcam; 1:1000), anti-HPRT1 (Thermo Fisher Scientific; 1:500), anti-viperin (Hycult Biotech; 1:500). Secondary/tertiary antibodies used were goat anti-rabbit HRP (VectorLabs; 1:1000), rabbit anti-chicken HRP (Thermo Fisher Scientific; 1:1000), and horse anti-mouse HRP (VectorLabs; 1:1000). Chemiluminescent signal was developed using either Novex CDP-Star kit for alkaline phosphatase (Thermo Fisher Scientific) or WesternBright Quantum kit for horseradish peroxidase (Advansta; #K-12042-D20). The signal was subsequently quantified using Fiji software [46]. For stripping of antibodies, membranes were incubated with stripping solution (62.5 mM Tris HCl pH 6.8, 2% SDS, 0.8% β-mercaptoethanol) for 45 minutes at 50°C. Subsequently, membranes were extensively washed six times with PBS. Following this, membranes were blocked, and immunostaining was again performed as described above.

### Luciferase assay

For analyses of *Renilla* luciferase activity in CHX-treated cells, *Renilla* Luciferase Assay Kit from Promega (#E2810) was used according to the manufacturer’s instructions. Briefly, 5×10^4^ DAOY cells per well were seeded on a 96-well plate. Cells were transfected with 100 ng of pRL-CMV vector per well using PolyJet transfection reagent and incubated with cycloheximide (50–300 μg/ml) for 2, 4, 6, 14, and 24 hours. At 24 hours post-transfection, the viability of cells was measured using alamarBlue. Subsequently, cells were lysed and *Renilla* luciferase activity was determined.

### Metabolic labelling of *de novo* synthesised proteins

Cells were seeded in 6-well plates at a density of 1×10^6^ (Vero, A549) or 5×10^5^ (DAOY, MG-63) cells per well. At indicated time intervals p.i., cells were washed with PBS and starved for 1 hour by addition of complete methionine-free RPMI medium (methionine-free RPMI medium containing 10% FBS, 1% L-alanyl-L-glutamine, 1% antibiotics/antimycotics, and 0.27 mM L-cystine). Subsequently, fresh complete methionine-free RPMI medium was added with 50 μM L-azidohomoalanine (AHA) and 1× AlamarBlue reagent. Metabolic labelling with AHA was performed for 2 hours. Afterwards, cell viability was measured as described earlier. Cells were then washed with PBS and lysed on ice for 15 minutes in 200 μl RIPA buffer with protease inhibitors (Halt Protease Inhibitor Single-Use Cocktail; Thermo Fisher Scientific). Lysates were separated on 12% polyacrylamide gels and transferred by electroblotting onto the PVDF membrane. The quantity of proteins loaded onto the gel was normalised to the cell numbers. Subsequently, the modified detection method Click-on-membrane was performed according to Kočová et al. (in preparation). Briefly, membranes were washed in 0.1 M potassium phosphate buffer pH 7.0 and the Click reaction was performed as follows: membranes were incubated in Click reaction buffer (0.1 M potassium phosphate buffer pH 7.0 with 0.25 mM sodium ascorbate, 0.5 mM THPTA, 0.1 mM CuSO_4_, and 10 μM biotin-alkyne) for 1 hour in the dark at room temperature. Membranes were washed three times with PBS, blocked (5% skimmed milk in PBS-T) and incubated with primary (AP-streptavidin; VectorLabs; 1:500), secondary (biotinylated anti-streptavidin; VectorLabs; 1:1000) and tertiary antibodies (AP-streptavidin; VectorLabs; 1:2000). Between each staining step, membranes were washed three times in PBS-T. Chemiluminescence signal was developed using Novex CDP-Star kit (Invitrogen; #WP20002). Signal was subsequently quantified using Fiji software [46].

### Metabolic labelling of *de novo* synthesised RNA

DAOY cells were seeded in 6-well plates at a density of 5×10^5^ cells per well. At the indicated time intervals p.i., 5-ethynyl uridine (5-EU) was added to the cells (final concentration of 5-EU was 1 mM) as well as alamarBlue reagent. Metabolic labelling with 5-EU was performed for 2 hours. Cell viability was measured as described earlier. Cells were then washed with PBS and lysed using RNA Blue reagent. Total RNA was isolated according to the manufacturer’s instructions. Next, RNA was separated in MOPS-buffered denaturing gel, as described above. The quantity of RNA was normalised to the cell number. Capillary blotting of RNA to the PVDF membrane (GE Healthcare) using 20× SSC buffering system was performed afterwards. Subsequently, the modified detection method Click-on-membrane was performed according to the method described by Kočová et. al. (in preparation). Briefly, the UV-fixed membrane was washed in 0.1 M potassium phosphate buffer pH 7.0 and the Click reaction on membrane was performed as follows: membranes were incubated in Click reaction buffer (0.1 M potassium phosphate buffer pH 7.0 with 0.25 mM sodium ascorbate, 0.5 mM THPTA, 0.1 mM CuSO_4_, and 10 μM picolyl biotin azide) for 1 hour in the dark at room temperature. Blocking and triple labelling using biotin-streptavidin system was performed as described above. The chemiluminescence signal was developed using Novex CDP-Star kit (Invitrogen; #WP20002), and signal was subsequently quantified using Fiji software [46].

### Immunofluorescence

DAOY cells were seeded in chamber slides (0,3 cm^2^/well; 5×10^3^ cells/well) and at the indicated time intervals p.i. processed as previously described [47]. Rabbit anti-POLR1A (Abcam; 1:200) and chicken anti-NS3 (a kind gift from Dr. M. Bloom, NIAID, NIH; 1:5000) antibodies were used. As the secondary antibodies, anti-rabbit DyLight 594 (Abcam; 1:500) and anti-chicken DyLight 488 (Abcam; 1:500), were used. In the case of metabolic labelling of nascent RNA, the Click reaction was performed *in situ* before the blocking step. 10 μM Picolyl biotin azide was used for the detection of incorporated 5-EU. For subsequent fluorescent labelling, streptavidin conjugated with DyLight 549 was used (VectorLabs; 1:500). Slides were eventually mounted in Vectashield mounting medium (VectorLabs). The Olympus Fluoview FV10i confocal microscope was used for imaging and subsequent export of images was done in FV10-ASW software (v.1.7).

### Statistical analyses

All statistical analyses were performed in MS Excel using one-sample two-tailed Studentʼs t-test. Only in case of qPCR analysis of over-expressed viperin and GFP, an unpaired two-tailed Student’s t-test was used. In this case, datasets were first tested for the equality of variances by F-test. If the experiment was performed in technical replicates, the statistics was performed using the means of the independent biological replicates.

## Results

### TBEV infection reduces host protein production

Recent studies have shown that DENV decreases the rate of *de novo* protein synthesis in host cells [24, 48]. In order to establish whether TBEV also affects translation, *de novo* protein synthesis kinetics was measured in TBEV-infected cells using Click chemistry [49]. For this purpose, we utilized a suitable *in vitro* experimental system of the cerebellum-derived human medulloblastoma cell line DAOY HTB-186 to broaden previous findings [47]. Two closely related members of the European subtype of TBEV with different virulence were used for comparative purposes: a medium virulent prototype strain, Neudoerfl, and a highly virulent strain, Hypr [41]. Initially, we characterized the course of infection for both TBEV strains. DAOY cells were infected at an MOI of 5 with either strain and at 12, 18, 24, 36, 48 hours p.i., replication kinetics, infection rate, viral protein (C, NS3) production and viability of infected cells were determined. Both strains successfully replicated in DAOY cells, with the Hypr strain reaching at least one order of magnitude higher titres during the course of infection until 48 hours p.i., when both strains eventually produced equal titres (Fig 1A). The infection rate was also considerably higher for the Hypr strain, culminating at 36 hours p.i. (87.5% of infected cells), whereas the Neudoerfl strain infected only 43.6% of cells (Fig 1A). Relative quantification of genomic RNA at 24 and 48 hours p.i. revealed that Hypr replicated with higher efficiency than Neudoerfl (Fig 1B). TBEV C and TBEV NS3 protein detection corresponded to replication kinetics and for both strains proteins could be detected earliest at 18 hours p.i., increasing thereafter (Fig 1C). While TBEV Neudoerfl affected the viability of the infected cells only mildly (maximal decrease by 16.6% at 36 hours p.i.), TBEV Hypr lowered the viability of the infected cells by 23.8% and 62.5% in comparison to mock-infected control at 36 and 48 hours p.i., respectively (Fig 1D). Therefore, in order to compensate the potential bias originating from cell death, we standardised our experiments to viability which is directly proportional to the number of living cells (S1 Fig). In the following experiments we pursued interaction of TBEV with DAOY cells during the period of productive infection for both TBEV strains, ranging from 24 to 48 hours p.i.

**Fig 2:**
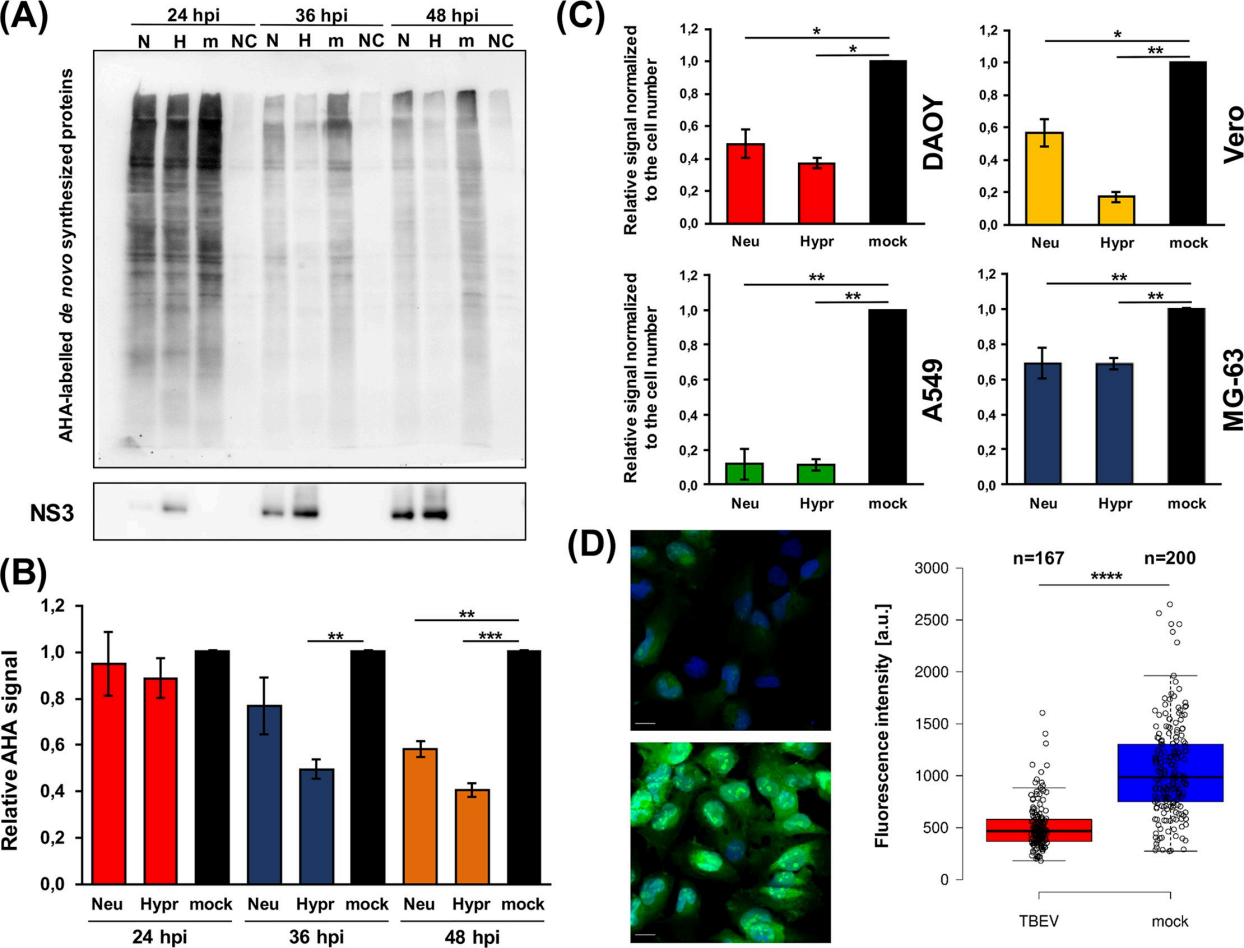
TBEV infection induces host translational shut-off. (A) DAOY cells were infected with TBEV Neudoerfl or Hypr strain (MOI 5) and *de novo* protein synthesis was assessed at 24, 36, and 48 hours p.i. by incorporation of methionine analogue L-azidohomoalanine (AHA). AHA-labelled proteins were visualised by immunodetection using HRP-conjugated antibodies; stripped membranes were subsequently used for the immunodetection of viral NS3 protein. Data are representative of three independent experiments; N–TBEV Neudoerfl strain (AHA-labelled), H–TBEV Hypr strain (AHA labelled), m–mock (AHA-labelled), NC–negative control (non-labelled). (B) Summary of *de novo* protein synthesis from (A) including all three performed experiments. Relative chemiluminescent signal was quantified using Fiji software and compared to mock-infected cells. Values were further normalised to the cell number and mock-infected cells were set to 1. Data are representative of three independent experiments and values are expressed as mean with SEM; significant difference from control was calculated by unpaired Student’s t-test (** P<0.01; *** P<0.001). (C) Summary of *de novo* protein synthesis rate in TBEV-infected DAOY, Vero, A549, and MG-63 cells. Cell lines were infected with either Neudoerfl or Hypr strain (MOI 5) and subsequently analysed for *de novo* protein synthesis at 48 hours p.i. Relative chemiluminescent signal was quantified using Fiji software and compared to mock-infected cells. Values were further normalised to the cell number and mock-infected cells were set to 1. Data are summary of three independent experiments and values are expressed as mean with SEM; significant difference from mock-infected cells was calculated by Student’s t-test (* P<0.05; ** P<0.01). (D) DAOY cells were infected with TBEV Hypr strain (MOI 5), and *de novo* protein synthesis was assessed at 36 hours p.i. by incorporation of methionine analogue L-azidohomoalanine (AHA). AHA-labelled proteins were visualised by Click reaction using AlexaFluor 488-conjugated alkyne. Representative images of TBEV-infected and control cells are shown on the left. Scale bar represents 100 μm. On the right, scatter plot is shown illustrating *de novo* protein synthesis rate measured by fluorescence intensity of the AlexaFluor 488 (fluorescence intensity per pixel; a.u.–arbitrary units). Data are representative of two independent experiments and values in graphs are expressed as mean, with whiskers extending to data points that are less than 1.5 x interquartile range away from 1^st^/3^rd^ quartile (Tukey’s boxplot); significant difference from mock-infected cells was calculated by Student’s t-test (**** P<0.0001).

After this detailed characterization of our *in vitro* model, *de novo* protein synthesis and quantification was performed. DAOY cells were infected with either TBEV Hypr or Neudoerfl and metabolic labelling was carried out for 2 hours at 24, 36, and 48 hours p.i. using the methionine analogue L-azidohomoalanine (AHA). At 24 hours p.i., translation levels were comparable in control and infected cells, but infection resulted in a significant decrease of AHA-labelled proteins at 36 and 48 hours p.i. in TBEV Hypr-infected cells and at 48 hours p.i. in TBEV Neudoerfl-infected cells (Fig 2A and 2B; S2A Fig). Interestingly, the viral NS3 protein levels increased over the course of the infection with both strains (Fig 2A, lower panel). Furthermore, TBEV-induced host translational shut-off was also documented for cell lines of non-neural origin (A549 cells, Vero cells, and MG-63 cells) at 48 hours p.i., for both TBEV strains (Fig 2C; S2B Fig). Interestingly, despite the observed host translational shut-off both TBEV strains were able to replicate (Fig 1B) successfully and reached high titres (Fig 1A) in DAOY cells throughout the infection.

**Fig 3:**
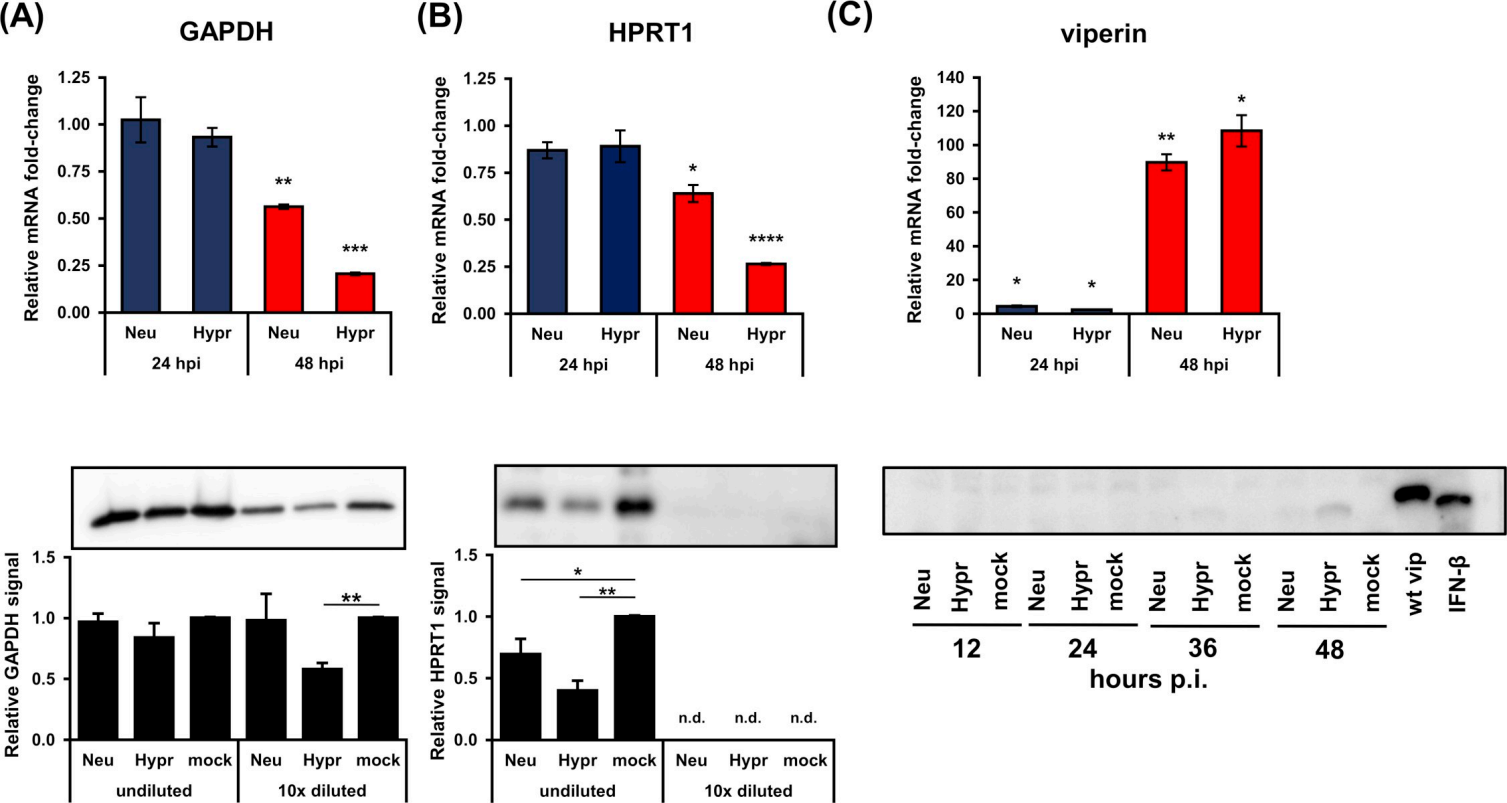
TBEV-induced translational arrest results in the decreased protein levels of GAPDH, HPRT1, and viperin. (A) Upper panel: DAOY cells were infected with either TBEV Neudoerfl or Hypr strain (MOI 5) and total RNA was isolated at indicated time intervals. Relative qPCR quantification of GAPDH mRNA using Δ-c_t_ method with normalisation to the cell number was performed. Lower panel: DAOY cells were infected with either Neudoerfl or Hypr TBEV strain (5 MOI) and lysed at 48 hours p.i. Western blot analysis of GAPDH protein levels was performed using protein-specific antibodies with undiluted and 10-times diluted samples. Relative chemiluminescent signal was quantified using Fiji software and compared to mock-infected cells. Values were further normalised to the cell number. Data are summary of three independent experiments and values in graphs are expressed as mean with SEM. Significant difference from mock-infected cells was calculated using one-sample Student’s t-test (** P<0.01; *** P<0,001). (B) Upper panel: DAOY cells were infected with either TBEV Neudoerfl or Hypr strain (MOI 5) and total RNA was isolated at indicated time intervals. Relative qPCR quantification of HPRT1 mRNA using Δ-c_t_ method with normalisation to the cell number was performed. Lower panel: DAOY cells were infected with either Neudoerfl or Hypr TBEV strain (5 MOI) and lysed at 48 hours p.i. Western blot analysis of HPRT1 protein levels was performed using protein-specific antibodies with undiluted and 10-times diluted samples. Relative chemiluminescent signal was quantified using Fiji software and compared to mock-infected cells. Values were further normalised to the cell number. Data are summary of three independent experiments and values in graphs are expressed as mean with SEM. Significant difference from mock-infected cells was calculated using one-sample Student’s t-test (* P<0.05; ** P<0.01; **** P<0,0001); n.d.–not detected. (C) Upper panel: DAOY cells were infected with either TBEV Neudoerfl or Hypr strain (MOI 5) and total RNA was isolated at the indicated time intervals. Relative qPCR quantification of viperin mRNA using Δ-c_t_ method with normalisation to the cell number was performed. Data are summary of three independent experiments and values are expressed as mean with standard error of mean (SEM). Lower panel: Immunodetection of viperin protein in TBEV-infected DAOY cells at indicated intervals p.i. (MOI 5). As a positive control, cells transfected with a c-myc-tagged viperin expression plasmid (wt vip) and cells treated with IFN-β (12 hours; 50 ng/ml) were used.

Since these experiments revealed a significant decrease in host protein synthesis upon TBEV infection on a global level, we evaluated the specificity of this for particular host proteins. First, the effect of TBEV infection on common housekeeping genes GAPDH and HPRT1 was determined by analysing their mRNA and protein levels. Relative quantification of GAPDH and HPRT1 mRNAs revealed a strong inhibition of expression for both genes and TBEV strains at 48 hours p.i. (Fig 3A and 3B; upper panel). Similar results were observed for their protein levels, although the more virulent strain Hypr elicited a stronger reduction (Fig 3A and 3B; lower panel).

As the subversion of host translation process can be used as an immune evasion strategy by viruses [17], we investigated the effect of translational shut-off on the interferon-inducible gene viperin. Viperin has been described so far as an antiviral protein that interferes with TBEV on multiple levels [50]. A time course of viperin mRNA production in response to TBEV infection in DAOY cells was determined. Induction of viperin mRNA expression was detected at 24 hours p.i. and increasing throughout next 24 hours (Fig 3C; upper panel). Despite significantly increased viperin mRNA levels, none or very small amounts of viperin protein were detected in cell lysates from TBEV-infected DAOY cells by western blot analysis (Fig 3C; lower panel). As a positive control, DAOY cells treated with INF-β (12 hours; 50 ng/ml) as well as DAOY cells transfected with a human viperin expression vector [40] were used.

To assess whether the effect of TBEV on endogenous viperin production can be overcome by artificial over-expression, DAOY cells were first infected (TBEV Neudoerfl and Hypr; MOI 5) and subsequently transfected with a wt-viperin expression construct at 12 hours p.i. Viperin mRNA, as well as protein levels, were analysed at 12 hours post-transfection (S3A Fig). As a control, GFP expression construct was used. S3B Fig shows that viperin protein was produced; however, the protein levels were significantly reduced in TBEV-infected cells compared to control cells. Hypr strain infection also resulted in a statistically significant decrease in mRNA levels of viperin. As expected, GFP production in TBEV infected cells was negatively affected in case of both TBEV strains (S3C Fig). Again, Hypr strain infection also caused a significant decrease in GFP mRNA. Consequently, TBEV induces a general translational shut-off, which can negatively affect even the production of overexpressed transcripts. Nevertheless, viral titres were increasing throughout the infection (Fig 1).

### TBEV infection downregulates the levels of specific host rRNAs

**Fig 4:**
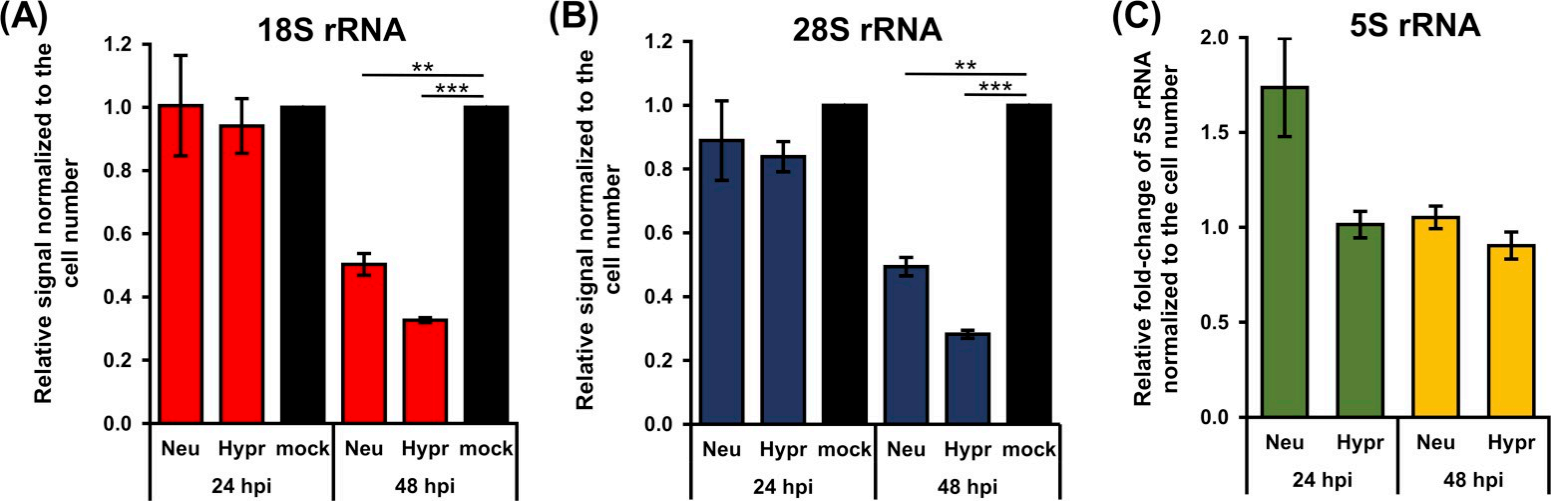
TBEV infection decrease levels of 18S and 28S rRNA but not 5S rRNA. (A, B) Total RNA was isolated from TBEV-infected DAOY cells (24 and 48 hours p.i.; MOI 5) and analysed using Bioanalyzer 2100. Graphs represent relative mean of areas for 18S (a) and 28S (b) peaks compared to mock-infected cells. Values were further normalised to the cell number. Data are representative of three independent experiments and values are expressed as mean with SEM. Significant difference from mock-infected cells was calculated using one-sample Student’s t-test (** P<0.01; *** P<0,001). (C) Relative quantification of 5S rRNA in TBEV-infected DAOY cells at 24 and 48 hours p.i. (MOI 5) using the Δ-c_t_ method. Graph represents relative fold-induction of 5S rRNA levels in comparison to mock-infected cells with normalisation to cell number. Data are representative of three independent experiments and values are expressed as mean with SEM.

Previous data revealed a significant decrease in RNA encoding genes including 5.8S rRNA and 7SL RNA following TBEV infection [47]. Here, we verified the link between the TBEV-induced translational shut-off and production of host rRNAs. We quantified the levels of 18S and 28S rRNAs in total cellular RNA from TBEV-infected DAOY cells at 24 and 48 hours p.i. We found that infection by both TBEV strains significantly decreased the 18S and 28S rRNA (S4 Fig). 18S rRNA levels decreased to 50 ± 6% or 33 ± 1% for TBEV Neudoerfl- or Hypr-infected cells compared to controls, respectively (Fig 4A). For 28S rRNA, its transcription levels fell to 49 ± 5% or 28 ± 2% for TBEV Neudoerfl- or Hypr-infected cells, respectively (Fig 4B). Both 18S and 28S rRNAs are transcripts of POLR1. Interestingly, the POLR3 transcript 5S rRNA levels remained unaffected by TBEV infection (Fig 4C). These data imply that the effect of TBEV infection on host cells also involves the transcription of specific ribosomal RNA genes.

### TBEV interferes with *de novo* production of 45-47S pre-rRNA transcripts

**Fig 5:**
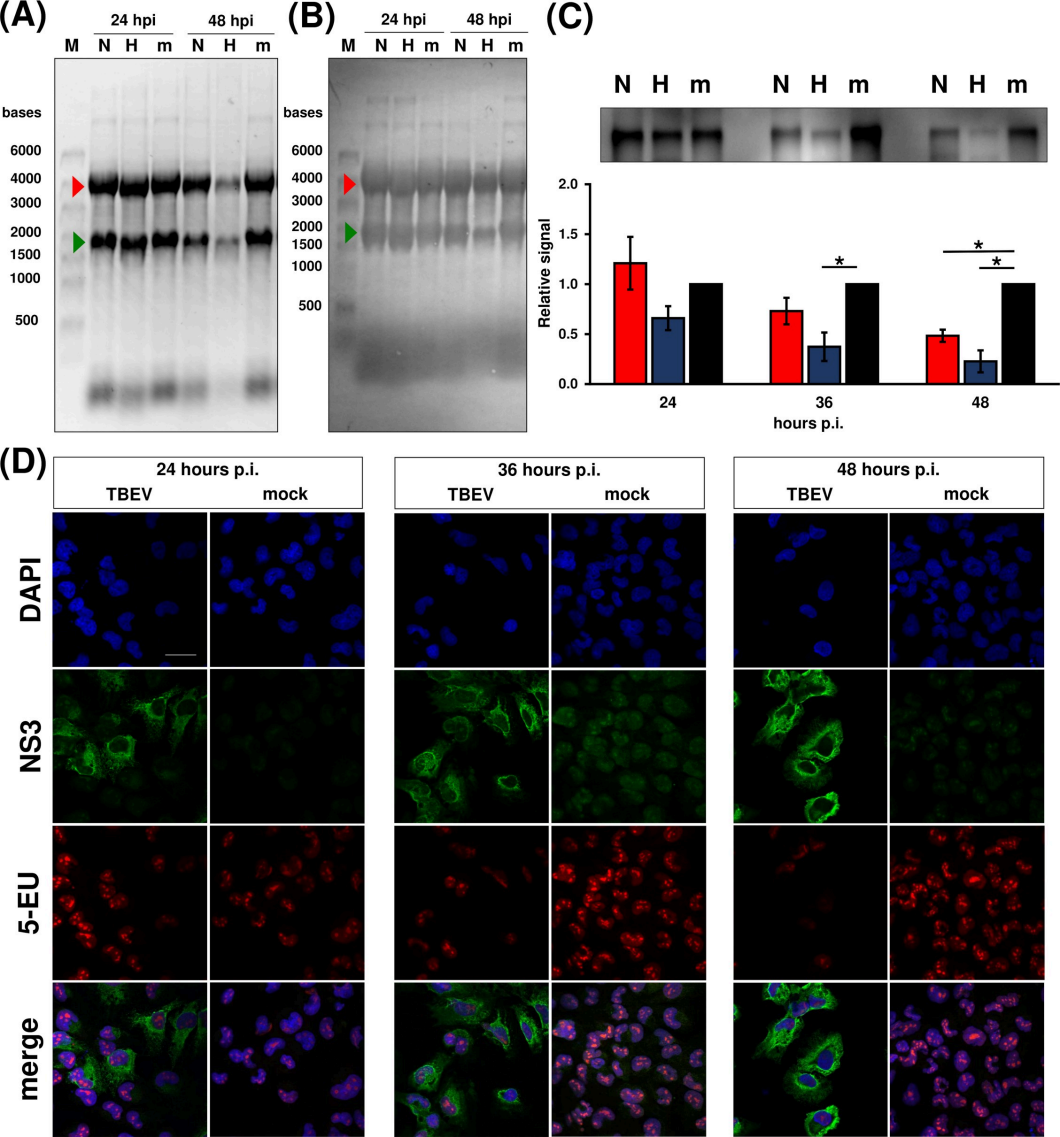
TBEV infection results in decrease of *de novo* synthesised 45-47S pre-rRNA. DAOY cells were either infected with Neudoerfl (N) or Hypr strain (H) at MOI 5 or mock-infected (m). Total RNA was isolated at indicated time post infection; 5-ethynyl uridine (1 mM) was added 2 hours before the collection interval. Data are representative of three independent experiments. (A) Integrity of 28S (red arrow) and 18S rRNA (green arrow), evaluated by using in-gel staining with GelRed. (B) Integrity of 28S (red arrow) and 18S rRNA (green arrow), evaluated by methylene blue staining after capillary transfer on PVDF membrane. (C) Upper panel: metabolic labelling of nascent 45-47S pre-rRNA was carried out using Click chemistry and biotin picolyl azide (10 μM) with subsequent chemiluminescent visualisation via biotin-streptavidin-alkaline phosphatase system. Lower panel: values are expressed as mean of three independent experiments with SEM. Significant difference from mock-infected cells was calculated using one-sample Student’s t-test (* P<0.05). (D) *In situ* metabolic labelling revealed TBEV-induced reduction of nascent RNA at 36 hours p.i. without change in RNA localization. DAOY cells were infected with TBEV Hypr strain (MOI 5) and at indicated time intervals incubated for 2 hours with 1 mM 5-ethynyl uridine (5-EU) in order to label nascent RNA. Detection of incorporated 5-EU was performed by Click reaction using 10 μM biotin picolyl azide followed by fluorescent labelling with streptavidin-DyLight549. Cells were co-stained with anti-NS3 antibodies; signal was further visualised using anti-chicken DyLight488 antibodies. Nuclei were stained with DAPI. Scale bar represents 200 μm.

In order to elucidate at which step TBEV interferes with rRNA production, we first analysed the integrity of mature rRNA molecules. No degradation products were observed following infection with either TBEV strains at 24 or 48 hours p.i. in DAOY cells (Fig 5A and 5B). Next, we investigated the rRNA expression and processing via quantification of *de novo* synthesised RNA in TBEV-infected DAOY cells. We labelled nascent RNA in TBEV-infected DAOY cells at 24, 36 and 48 hours p.i. with 5-ethynyl uridine (EU). Incorporated EU was visualised using Click chemistry and the biotin-streptavidin detection system. The presence of TBEV Hypr strain resulted in a decreased quantity of 45-47S pre-rRNA transcripts at 36 and 48 hours p.i., whereas infection with TBEV Neudoerfl strain reduced *de novo* synthesis of 45-47S pre-rRNA at 48 hours p.i. (Fig 5C).

Previously, a link between the inhibition of expression of 45-47S pre-rRNA and nucleolar stress was documented [31]. There are several hallmarks typical for nucleolar stress including disruption of nucleolus structure [51]. We, therefore, characterised the localization and production of nascent RNA at the cellular level and also investigated the structure of the nucleolus. DAOY cells infected with TBEV Hypr strain were analysed at 24, 36, and 48 hours p.i. using *in situ* Click reaction with 10 μM picolyl biotin azide and subsequent visualisation *via* streptavidin conjugated with DyLight-549. As shown in Fig 5D, the overall production of nascent RNA in TBEV-infected cells started to decrease from 36 hours p.i.; *de novo* synthesised RNA was exclusively detected in nuclei with foci of nascent RNA molecules localised in nucleoli. In addition, these nascent RNA foci were not structurally altered upon TBEV infection. The specificity of the labelling reaction was determined using EU-unlabelled cells in the Click reaction (S5A Fig). In order to further verify that TBEV did not induce nucleolar re-arrangement due to nucleolar stress, we analysed the nucleolar structure upon TBEV Hypr infection using nucleophosmin (NPM1; a nucleolar marker). As a positive control, cells were treated with 1 mM H_2_O_2_ for 45 minutes. No disruption of nucleoli in TBEV-infected cells was observed (S5B Fig). These data imply that TBEV inhibits 45-47S pre-rRNA production without triggering the nucleolar stress pathway.

### TBEV infection affects POLR1 levels but not nucleolar localisation

**Fig 6:**
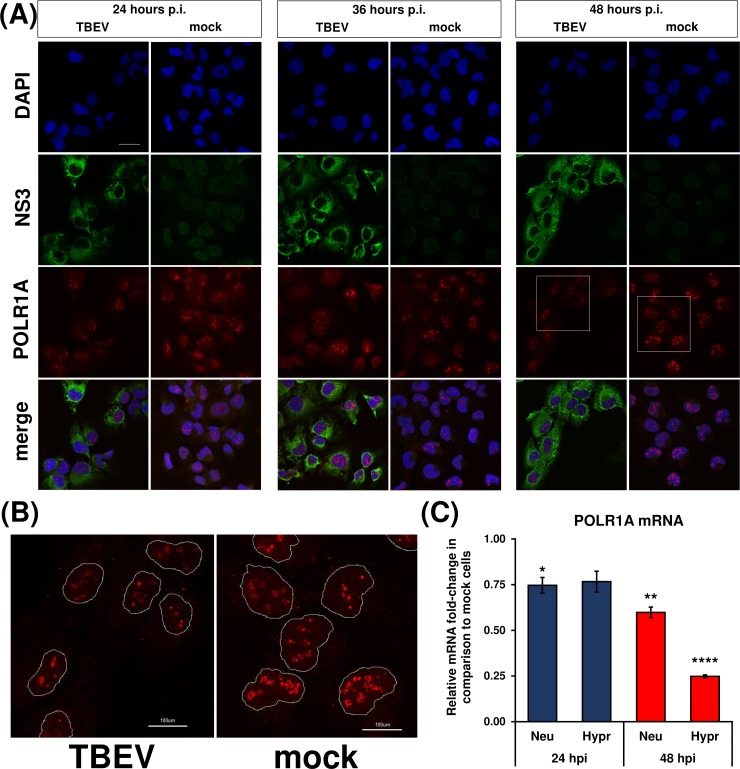
RNA polymerase I is not translocated upon TBEV infection. (A) DAOY cells were infected with TBEV Hypr strain (MOI 5) and at indicated time intervals fixed and POLR1A was detected using rabbit anti-POLR1A and anti-rabbit DyLight594 antibodies. Cells were further co-stained for viral NS3 protein using chicken anti-NS3 and anti-chicken DyLight488 antibodies. Nuclei were stained with DAPI. Scale bar represents 200 μm. (B) Zoomed images from panel (A) at 48 hours p.i. (areas marked by the white squares); POLR1 is localised in distinct foci in host nuclei without any observable virus-induced translocation. Scale bar represents 100 μm. (C) DAOY cells were infected with either TBEV Neudoerfl or Hypr strain (MOI 5) and total RNA was isolated at indicated time intervals. Relative qPCR quantification of POLR1A mRNA using Δ-c_t_ method with normalisation to the cell number was performed. Data are representative of three independent experiments and values are expressed as mean with SEM. Significant difference from mock-infected cells was calculated using one-sample Student’s t-test (* P<0.05; ** P<0.01; **** P<0,0001).

Based on the observed TBEV interference with rRNA production on the transcriptional level, we sought to investigate if the levels and cellular localization of POLR1 changes in infected cells. As shown in Fig 6A and 6B, POLR1 was localised exclusively to the nuclei, and no translocation occurred in infected cells at any time interval tested. Nevertheless, POLR1 protein levels were impaired in TBEV Hypr-infected cells at 48 hours p.i. This may be a result of the previously mentioned translational shut-off since it coincided at 48 hours p.i. Besides, POLR1 mRNA levels were negatively affected by TBEV infection, too (Fig 6C). In particular, POLR1A (the largest subunit of the RNA polymerase I complex) mRNA levels dropped to 60 ± 5% or 25 ± 1% in TBEV Neudoerfl- or Hypr-infected DAOY cells at 48 hours p.i., respectively.

**Fig 7:**
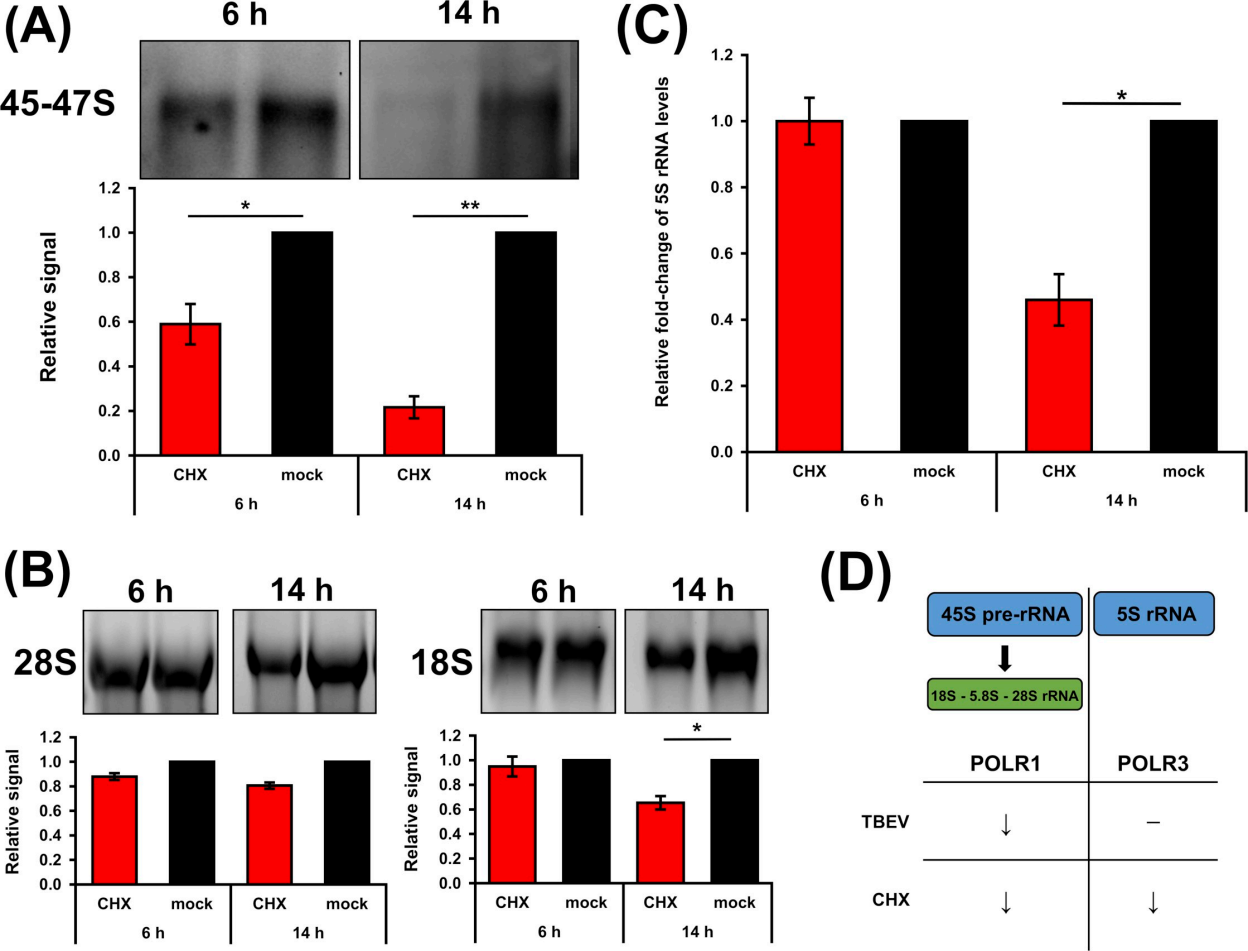
Cycloheximide (CHX) treatment decreases production of rRNA transcripts by POLR1 and POLR3. (A) DAOY cells were treated with CHX (100 μg/ml) for either 6 or 14 hours; for metabolic RNA labelling, 5-EU was added 2 hours before the sample collection. Cell viability was measured before the cell lysis. Isolated total RNA was transferred to a PVDF membrane and nascent RNA quantified using Click chemistry with 10 μM biotin picolyl azide before subsequent chemiluminescent detection. Data are representative of three independent experiments and values in graphs are expressed as mean with SEM, normalised to cell numbers and mock infected cells. Significant difference from the control was calculated using one-sample Student’s t-test (* P<0.05; ** P<0.01). (B) Levels of 28S and 18S rRNA were analysed by in-gel RNA staining with GelRed before blotting. Data are representative of three independent experiments and values in graphs are expressed as mean with SEM, normalised to cell number and mock infected samples. Significant difference from the control was calculated using one-sample Student’s t-test (* P<0.05). (C) Relative quantification of 5S rRNA in CHX-treated DAOY cells (6 and 14 hours post treatment; 100 μg/ml) using the Δ-c_t_ method. Graph represents relative fold-induction of 5S rRNA levels in comparison to mock-treated cells, with normalisation to cell number. Data are representative of three independent experiments and values are expressed as mean with SEM. Significant difference from the control was calculated using one-sample Student’s t-test (* P<0.05). (D) Schematic summary of CHX versus TBEV effect on the expression of POLR1 and POLR3 transcripts. (↓) indicates observed decrease of the RNA levels and (─) indicates no change in RNA levels.

TBEV-induced translational shut-off and the decrease in production of nascent 45-47S pre-rRNA raised the question whether these processes are casually interconnected. We analysed the rate of rRNA production in DAOY cells after treatment with cycloheximide (CHX), an inhibitor of translation elongation. First, we determined the time- and dosage-dependent effect of CHX in DAOY cells using a *Renilla* (RL) luciferase-based reporter system. DAOY cells were first transfected with pRL-CMV and treated with CHX (50, 100, and 300 μg/ml). As shown in S6 Fig, all CHX concentrations tested decreased the production of luciferase. Moreover, the inhibition rate of luciferase production increased with longer exposure to CHX. Next, rRNA production in DAOY cells with decreased translational rate was assessed. Cells were treated with CHX (100 μg/ml) for 6 or 14 hours and *de novo* RNA synthesis in CHX-treated cells was subsequently determined. Fig 7A shows a statistically significant decrease in levels of nascent 45-47S pre-rRNA for both intervals. In particular, the levels decreased to 22 ± 9% or 56 ± 16% during CHX treatment for 14 or 6 hours, respectively. In addition, total levels of mature 18S and 28S rRNAs were quantified in CHX-treated cells. Significant decreases in 18S rRNA levels were observed after a 14-hour incubation (65 ± 9%; Fig 7B). 28S rRNA levels were reduced to 81 ± 4% compared to control cells; however, this effect was not statistically significant (Fig 7B). Quantification of 5S rRNA, a POLR3 transcript, revealed a statistically significant decrease even for this rRNA species after 14 hours of CHX treatment (46 ± 11%; Fig 7C). These data demonstrated that during translation inhibition induced by CHX, the quantity of rRNA transcripts of both RNA polymerases (POLR1 and POLR3) were decreased. In comparison to the general rRNA synthesis shut-down resulting from the action of CHX, TBEV infection induced only a decrease in POLR1 rRNA transcripts (Fig 7D). This suggests that TBEV infection specifically targeted POLR1, which may subsequently result in translational shut-off.

## Discussion

TBEV infection is spreading through Europe, resulting in increased numbers of TBEV cases and emergence in previously unaffected areas. TBEV is known to be able to cause neurological symptoms in some infected patients, though little is known about its interplay with neural cells. The molecular basis of damage to the CNS following TBEV infection is still not fully understood. So far, it seems that it is a complex phenomenon combining multiple factors including host immune system [52]. Therefore, understanding the TBEV interaction with target cells and detailed description of processes of viral or host response can help to reveal new targets and ideas on how to treat this disease more successfully. To what extent the outcome of these infection-induced processes is reflected on longer term sequelae remains unrevealed.

Metabolic labelling experiments demonstrated that TBEV infection interferes with the global *de novo* protein synthesis in infected cells. Surprisingly, the effect of translational arrest was so robust that even the protein levels of two commonly used housekeeping genes, GAPDH and HPRT1, were significantly lowered (Fig 3A and 3B). Cell lines of both neural and non-neural origin underwent translational shut-off, demonstrating thus the general nature of this phenomenon upon TBEV infection. However, the rate of reduction varied substantially in individual cell lines suggesting cell-dependent effects. TBEV Hypr strain caused a greater translational shut-off in all cell lines compared to the Neudoerfl strain. This may be due to the increased virulence and neuroinvasiveness of the Hypr strain [53] or due to susceptibility and tropism of the virus strains to specific cell types. Recent studies have demonstrated that some flaviviruses can cause translation suppression via diverse mechanisms [24, 48]. These findings together with our results revise the idea of flaviviruses as “non-host cell protein synthesis influencers” [25, 54, 55]. Indeed, flaviviruses have been thought to avoid the host-cell protein synthesis shut-off as they replicate at a slower rate and global protein synthesis manipulation might have potentially deleterious effects on cell viability and virus yields [56, 57]. However, reduced synthesis of host proteins had no adverse effect on the production of viral NS3 and C proteins (Fig 1C), viral gRNA (Fig 1B) or production of viral progeny (Fig 1A). This suggests that protein synthesis shut-off does not stop TBEV from successful replication.

Viperin is a known interferon-stimulated gene (ISG) and has been described as a potent antiviral protein against members of the *Flaviviridae* family, especially TBEV [50, 58–61]. Thereby it is anticipated to see an increase in viperin mRNA levels upon TBEV infection in DAOY cells. However, the absence of endogenous viperin protein in TBEV-infected cells is surprising. Thus, translational shut-off may yield multiple advantages to TBEV. Apart from gearing the host protein synthesis apparatus to the purposes of the virus, it may also perform as an immune evasion strategy by preventing ISG production. A widely used stable overexpression approach in an ISG/viperin study [59] might therefore mask the real interactions among flaviviruses and host cells during the infection. In general, our data highlight the importance of careful experimental design when studying virus-host interactions and ISG function specifically.

To our knowledge virus-driven reduction in host rRNA levels has not been described before for any flavivirus. Only scarce information is available regarding the virus-induced reduction of rRNA expression, production, and maturation. For example, murine hepatitis virus directly reduces the levels of mature 28S rRNA [62]; *Autographa californica* multiple nucleopolyhedrovirus was shown to decrease both, 18S and 28S rRNAs [63]. Additionally, over-expression of HIV Tat protein in *Drosophila melanogaster* led to the impairment of 45S pre-rRNA precursor processing [35]. Similarly, herpes simplex virus 1 decreased the rate of rRNA maturation despite unaltered levels of 45-47S pre-rRNA and unchanged POLR1 activity [32]. The reduction of rRNA levels can be associated with the induction of nucleolar stress, which is characterized by several hallmarks including nucleolar and ribosomal disruption eventually leading to the activation of the p53 signalling pathway. A possible link between flaviviral pathogenesis and nucleolar stress was suggested previously. DENV and ZIKV, but not WNV were shown to induce nucleolar stress in infected cells by disruption of nucleoli, which resulted in an increased rate of apoptosis via the p53 signalling cascade [64]. However, no disruption of nucleoli was observed in the case of TBEV-infected DAOY cells (S5B Fig), possibly not surprising as the TBEV infection specifically affects only the POLR1 activity.

**Fig 8:**
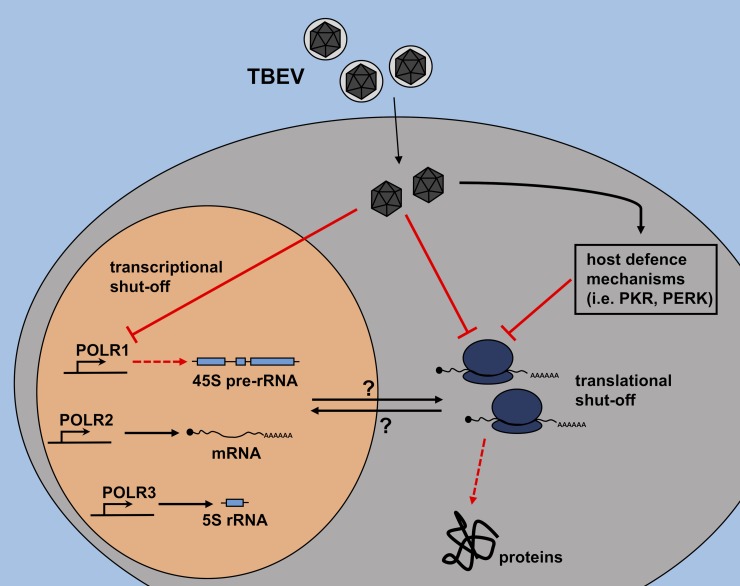
Schematic overview of potential pathways leading to TBEV-driven decrease in synthesis of host rRNA and proteins. TBEV may interfere directly with host translational processes, leading to decreased host protein levels. This decrease could negatively affect pre-rRNA synthesis and eventually rRNA levels. On the other hand, TBEV may also interfere directly with the synthesis of pre-rRNA first, which results in decreased levels of mature rRNAs. Insufficiency of rRNAs subsequently leads to the impairment of ribosome biogenesis and decrease of the translational rate in infected DAOY cells. TBEV infection could also trigger host defence mechanisms leading to the translational arrest. For example, protein kinase R (PKR) activated by dsRNA or PKR-like endoplasmic reticulum kinase (PERK) activated by ER stress could play a significant role in the observed translational shut-off as well.

We propose alternative ways by which TBEV could interfere with transcription and/or translation in DAOY cells: 1) TBEV negatively affects the translation of host proteins, including POLR1, transcription factors, and ribosomal proteins; their lower levels subsequently result in a decline in synthesis of all rRNA species; or 2) TBEV directly interferes with *de novo* synthesis of 45-47S pre-rRNA (but not 5S rRNA) via a POLR1 specific mechanism, which reduces the levels of 18S and 28S rRNAs and this leads to the decline of translational rate in host cells; 3) transcription and translation can be modified independently by both viral or cellular factors as a result of infection (summarised in Fig 8). Translational shut-off can otherwise be elicited by host cell defence mechanisms, such as activation of protein kinase R (PKR) or PKR-like endoplasmic reticulum kinase (PERK) [65–67]. To elucidate the exact mechanism of the inhibition of host protein and rRNA production and actual involvement of viral and host factors further experiments will be needed. These may for example assess whether viral proteins can directly inhibit transcription and/or translation. The present study does not elucidate this question and more work will be required to understand the processes; underlying the effects described here.

An overall translational inhibition induced by CHX treatment results in reduced *de novo* synthesis of 45-47S pre-rRNA precursor as well as the levels of 5S rRNA in DAOY cells. In contrast, TBEV infection only affected the 45-47S pre-rRNA precursor (and mature 18S and 28S rRNA levels) and did not affect 5S rRNA. This suggests TBEV-specific inhibition of POLR1 activity, which could result in reduced production of host proteins. Further analyses are needed to characterise the connection between rRNA production arrests and translational shut-off upon TBEV infection.

In summary, our results give new insights into the flavivirus-host interactions at the transcriptional/translational level. Moreover, a virus-induced rRNA decrease was described for flaviviral infection for the first time. The research here can contribute to understanding the mechanisms which determine at least to some extent the subsequent pathological processes. However, the relatively late onset of effects described in this study cannot completely rule out the possibility that our observations are due to cellular responses to TBEV infection rather than virus-mediated, or even combinations of both cellular and viral effects. More work is required to assess these possibilities in detail.

## Supporting information

S1 Fig Cell viability measurement using AlamarBlue in TBEV-infected DAOY cells. DAOY cells were infected with either TBEV Neudoerfl or Hypr strains (MOI 5) or untreated (mock); at indicated time intervals, cells were counted. A two-fold serial dilution was prepared with range from 50000 to 390 cells/well and cell viability was subsequently analysed by using alamarBlue reagent. Graphs represents fluorescent signal linked to the cell number at 24 hours p.i. (A) and 48 hours p.i. (B). Three independent experiments were performed and values are expressed as mean with SEM.
(TIF)

S2 Fig TBEV induces host translational shut-off in infected cells. (A) Total protein pattern visualized using Coomassie blue (CBB) staining of the gel used for AHA detection presented in Fig 2A. (N) TBEV Neudoerfl, (H) TBEV Hypr, (m) mock control, (NC) non-labelled mock control. (B) DAOY, MG-63, A549, and Vero cells were infected with either Neudoerfl (N) of Hypr (H) strains of TBEV (MOI 5); as a negative control, mock-infected (m) cells were included. Cells were starved for 1 hour in methionine-free medium and subsequently, nascent proteins were labelled using AHA (incubation for 2 hours; non-labelled negative controls, NC). Cell lysates analysed by SDS-PAGE followed by proteins transfer to PVDF membrane and Click reaction using biotin-alkyne. *De novo* synthesized proteins were further visualized by using biotin-streptavidin detection system along with conjugated alkaline phosphatase. Developed membranes were then stripped and NS3 viral protein detected. Total protein pattern was visualized using CBB staining of the gels after blotting. Representative images out of three independent experiments are shown.
(TIF)

S3 Fig TBEV inhibits production of over-expressed viperin and GFP. (A) Schematic overview of experimental procedure: DAOY cells were first infected with either Neudoerfl or Hypr strain (MOI 5) and at 24 hours p.i. transfected either with wt-viperin or phMGFP expression constructs. (B) The relative quantification of overexpressed viperin and GFP mRNA in either TBEV Neudoerfl- (Neu) or TBEV Hypr-infected DAOY cells at 24 hours p.t. The Δ-c_t_ relative quantification method was used, with normalisation to the cell number. Mock-transfected cells (empty vector only) were used as a control. Data are representative of three independent experiments and values are expressed as mean with SEM. Significant difference from the control was calculated using unpaired two-sample Student’s t-test (* P<0.05, ** P<0.01). (C) DAOY cells were first infected with either Neudoerfl or Hypr strain (MOI 5) and at 24 hours p.i. transfected with either viperin or GFP expression plasmids. Analysis of viperin and GFP protein levels was performed at 24 hours p.t. using viperin-specific immunodetection and GFP signal measurement. Relative amounts in comparison to uninfected cells with normalisation to cell numbers are shown for both proteins. Data are representative of three independent experiments and values are expressed as mean with SEM. Significant difference from the control was calculated using a one-sample Student’s t-test (* P<0.05).
(TIF)

S4 Fig Raw data of rRNA abundancy in TBEV-infected cells acquired from Bioanalyzer 2100. DAOY cells were infected with either TBEV Neudoerfl or Hypr strains (MOI 5) and total RNA was isolated with RNAblue at the indicated time intervals. Subsequent analysis was performed by using 30 ng of total RNA from mock-infected cells; RNA input of remaining samples was normalised to the cell number. Representative images from three independent experiments are shown.
(TIF)

S5 Fig Specificity of Click reaction and visualization of nucleoli in DAOY cells. (A) DAOY cells were infected with TBEV Hypr strain (MOI 5) and at indicated time intervals incubated for 2 hours with EU-free cultivation medium. Fixed cells underwent the Click reaction using 10 μM biotin picolyl azide followed by fluorescent labelling with streptavidin-DyLight549. Cells were co-stained with anti-NS3 antibodies; signal was further visualized using anti-chicken DyLight488 antibodies. Scale bar represents 200 μm. (B) DAOY cells were either infected with TBEV Hypr strain (MOI 5) and fixed at 48 hours p.i. or treated with 1 mM hydrogen peroxide for 45 minutes before the fixation. Anti-NPM1 antibodies with the secondary DyLight594-conjugated antibodies were used for the visualization of nucleoli. Scale bar represents 80 μm.
(TIF)

S6 Fig Cycloheximide (CHX) treatment results in decreased production of Renilla luciferase. DAOY cells were transfected with 100 ng of pRL-CMV reporter vector expressing RL and subsequently treated with CHX (50, 100 or 300 μg/ml) for time periods indicated. At 24 hours p.t. cell viability as well as luciferase activity was analysed. Data are representative of two independent experiments and values are expressed as mean with SEM.
(TIF)

S1 Table List of used primers. (PDF)
