# Supplementary figures and images for: Tick-borne encephalitis virus inhibits rRNA synthesis and host protein production in human cells of neural origin
Source: PLoS Negl Trop Dis. 2019 Sep 27;13(9):e0007745. doi: 10.1371/journal.pntd.0007745 (PMC6785130; doi:10.1371/journal.pntd.0007745)

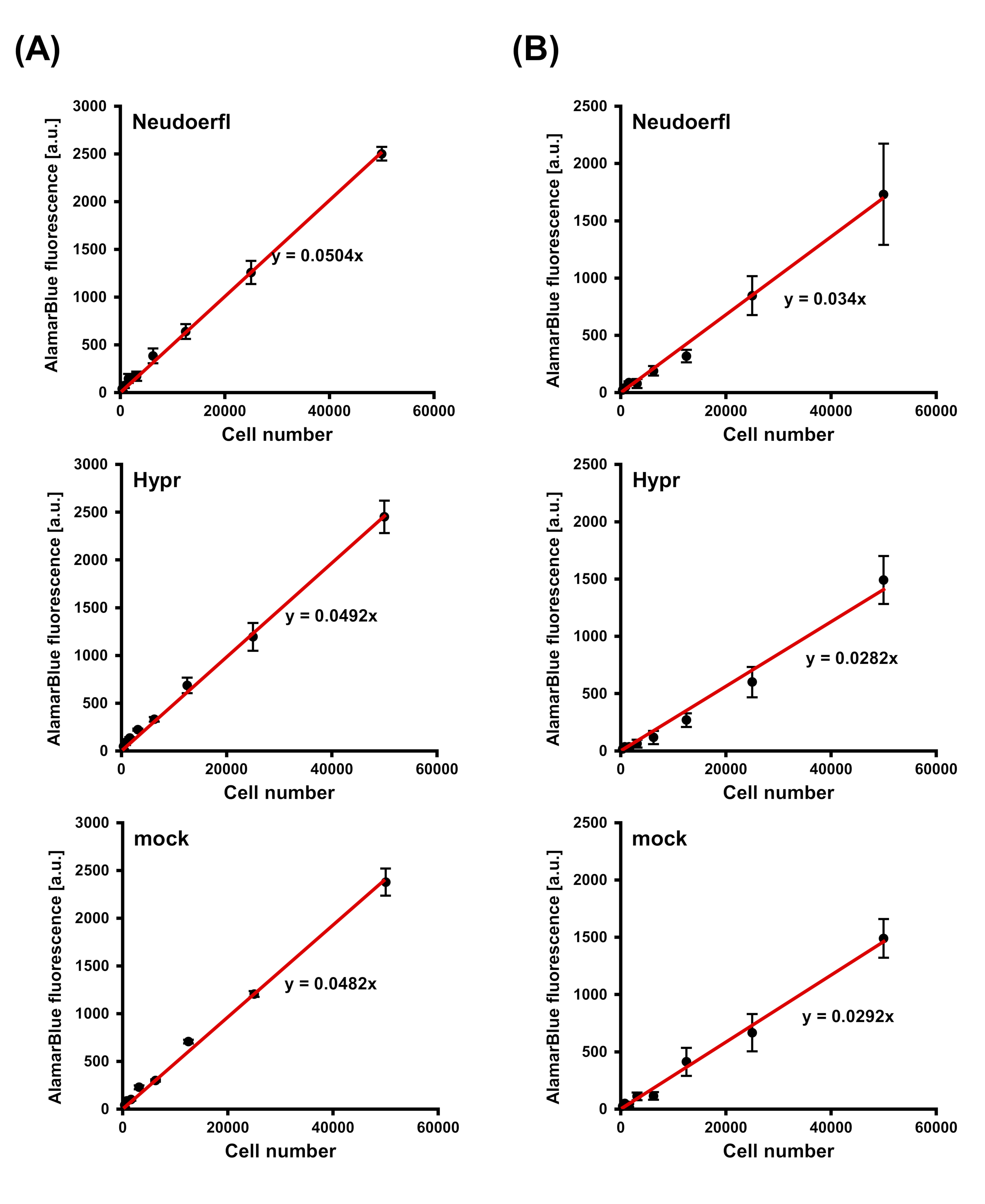

Supplement: S1 Fig — DAOY cells were infected with either TBEV Neudoerfl or Hypr strains (MOI 5) or untreated (mock); at indicated time intervals, cells were counted. A two-fold serial dilution was prepared with range from 50000 to 390 cells/well and cell viability was subsequently analysed by using alamarBlue reagent. Graphs represents fluorescent signal linked to the cell number at 24 hours p.i. (A) and 48 hours p.i. (B). Three independent experiments were performed and values are expressed as mean with SEM. (TIF) [file pntd.0007745.s001.tif]

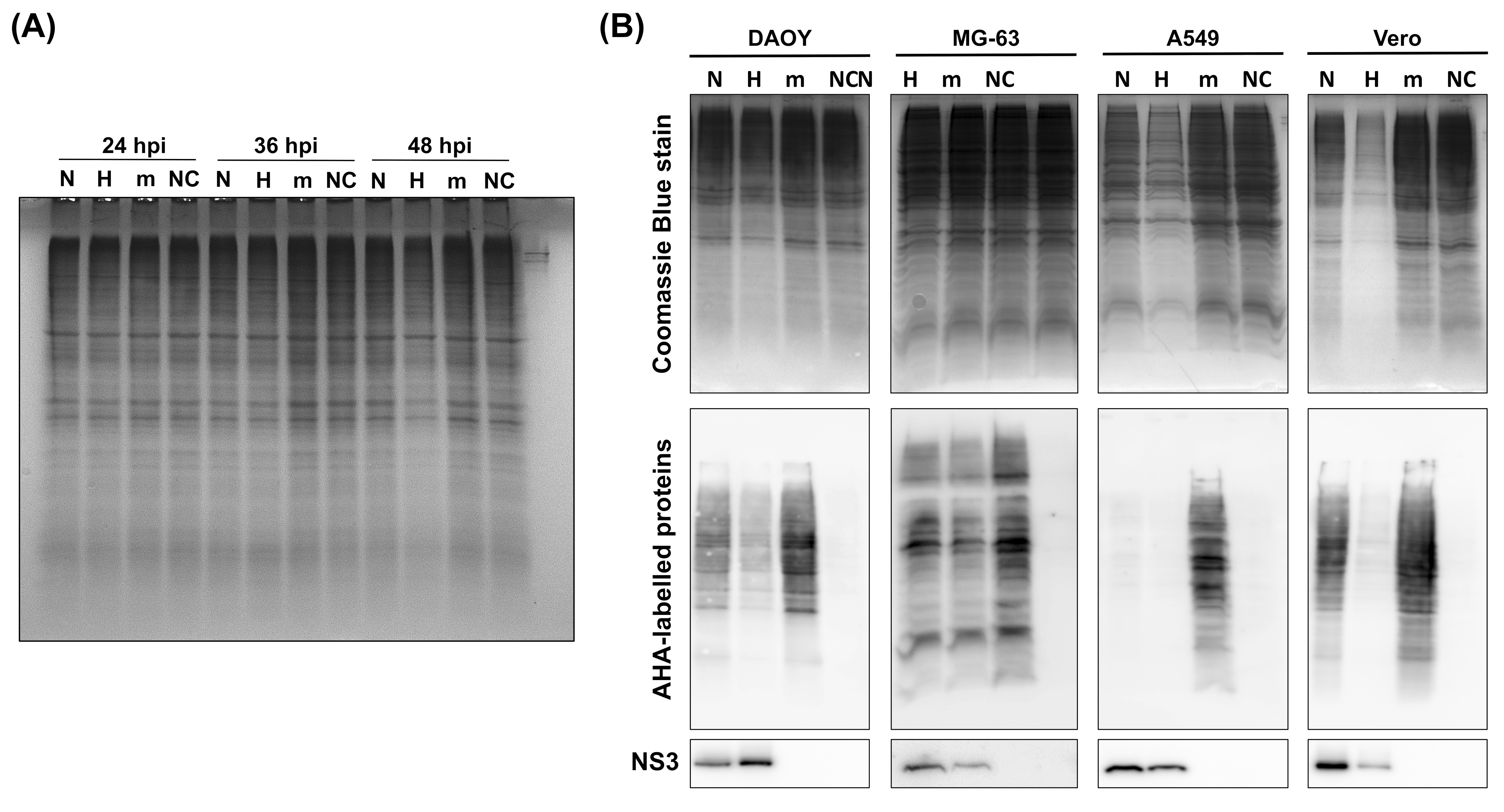

Supplement: S2 Fig — (A) Total protein pattern visualized using Coomassie blue (CBB) staining of the gel used for AHA detection presented in Fig 2A. (N) TBEV Neudoerfl, (H) TBEV Hypr, (m) mock control, (NC) non-labelled mock control. (B) DAOY, MG-63, A549, and Vero cells were infected with either Neudoerfl (N) of Hypr (H) strains of TBEV (MOI 5); as a negative control, mock-infected (m) cells were included. Cells were starved for 1 hour in methionine-free medium and subsequently, nascent proteins were labelled using AHA (incubation for 2 hours; non-labelled negative controls, NC). Cell lysates analysed by SDS-PAGE followed by proteins transfer to PVDF membrane and Click reaction using biotin-alkyne. De novo synthesized proteins were further visualized by using biotin-streptavidin detection system along with conjugated alkaline phosphatase. Developed membranes were then stripped and NS3 viral protein detected. Total protein pattern was visualized using CBB staining of the gels after blotting. Representative images out of three independent experiments are shown. (TIF) [file pntd.0007745.s002.tif]

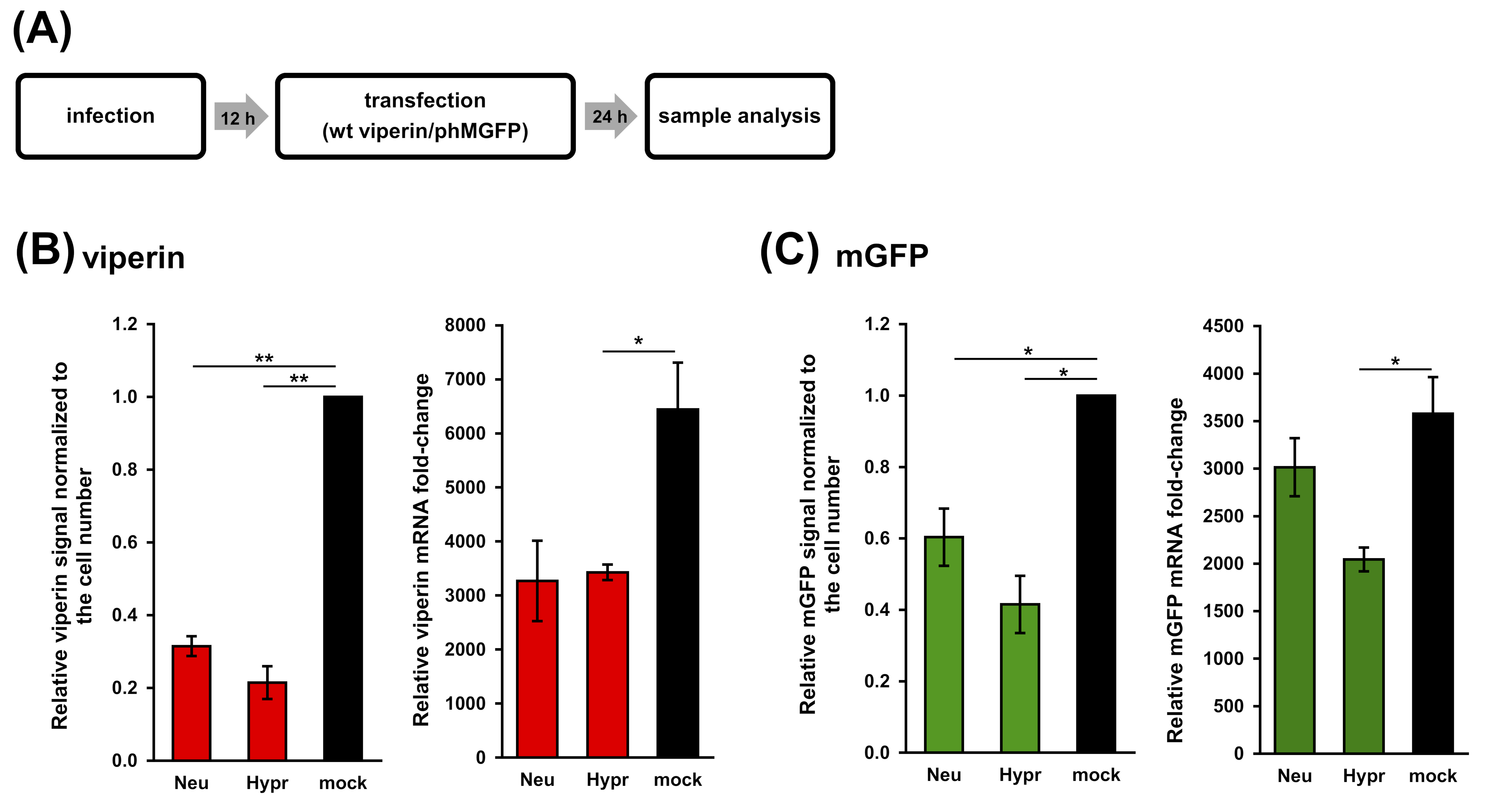

Supplement: S3 Fig — (A) Schematic overview of experimental procedure: DAOY cells were first infected with either Neudoerfl or Hypr strain (MOI 5) and at 24 hours p.i. transfected either with wt-viperin or phMGFP expression constructs. (B) The relative quantification of overexpressed viperin and GFP mRNA in either TBEV Neudoerfl- (Neu) or TBEV Hypr-infected DAOY cells at 24 hours p.t. The Δ-ct relative quantification method was used, with normalisation to the cell number. Mock-transfected cells (empty vector only) were used as a control. Data are representative of three independent experiments and values are expressed as mean with SEM. Significant difference from the control was calculated using unpaired two-sample Student’s t-test (* P<0.05, ** P<0.01). (C) DAOY cells were first infected with either Neudoerfl or Hypr strain (MOI 5) and at 24 hours p.i. transfected with either viperin or GFP expression plasmids. Analysis of viperin and GFP protein levels was performed at 24 hours p.t. using viperin-specific immunodetection and GFP signal measurement. Relative amounts in comparison to uninfected cells with normalisation to cell numbers are shown for both proteins. Data are representative of three independent experiments and values are expressed as mean with SEM. Significant difference from the control was calculated using a one-sample Student’s t-test (* P<0.05). (TIF) [file pntd.0007745.s003.tif]

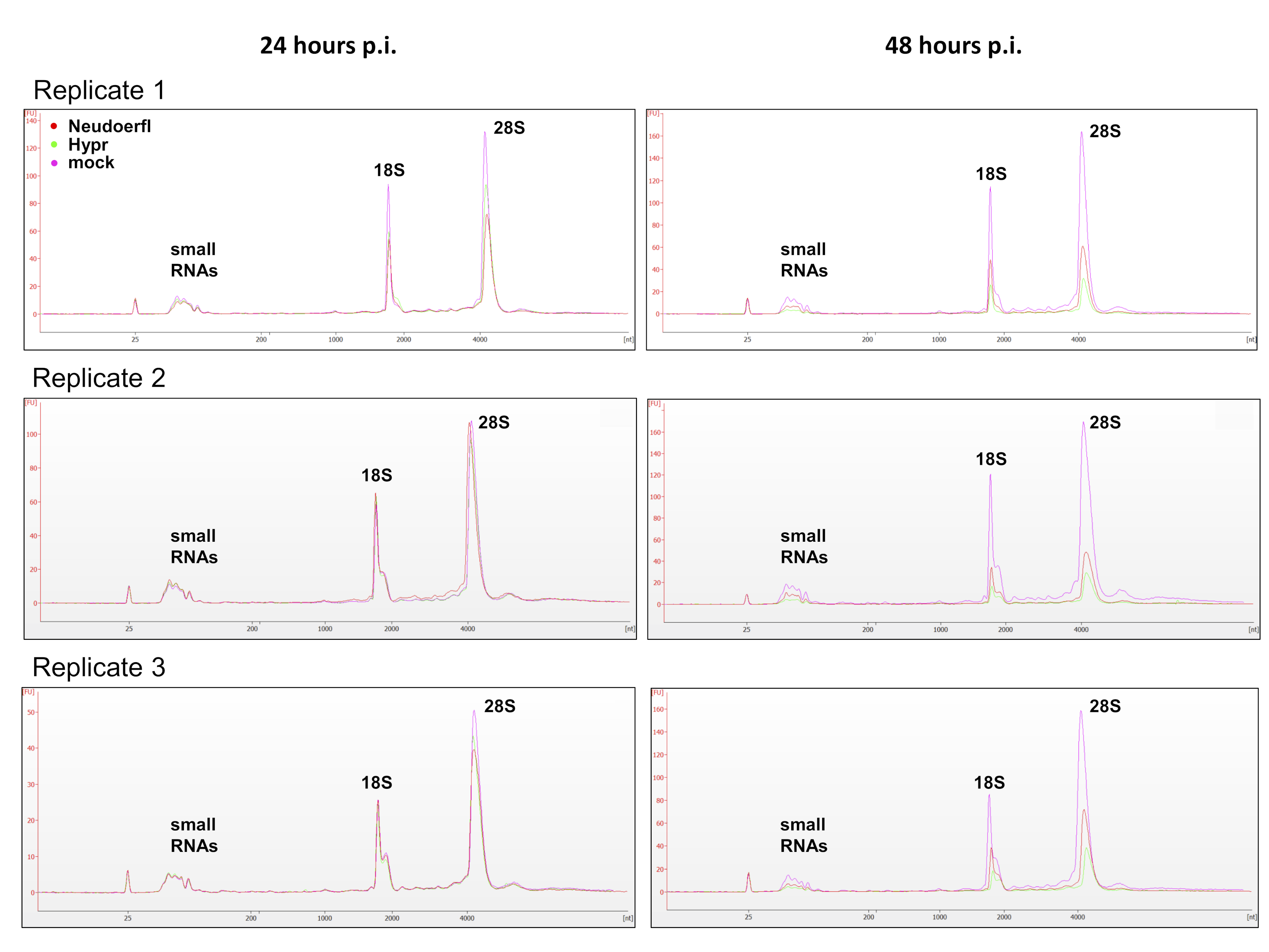

Supplement: S4 Fig — DAOY cells were infected with either TBEV Neudoerfl or Hypr strains (MOI 5) and total RNA was isolated with RNAblue at the indicated time intervals. Subsequent analysis was performed by using 30 ng of total RNA from mock-infected cells; RNA input of remaining samples was normalised to the cell number. Representative images from three independent experiments are shown. (TIF) [file pntd.0007745.s004.tif]

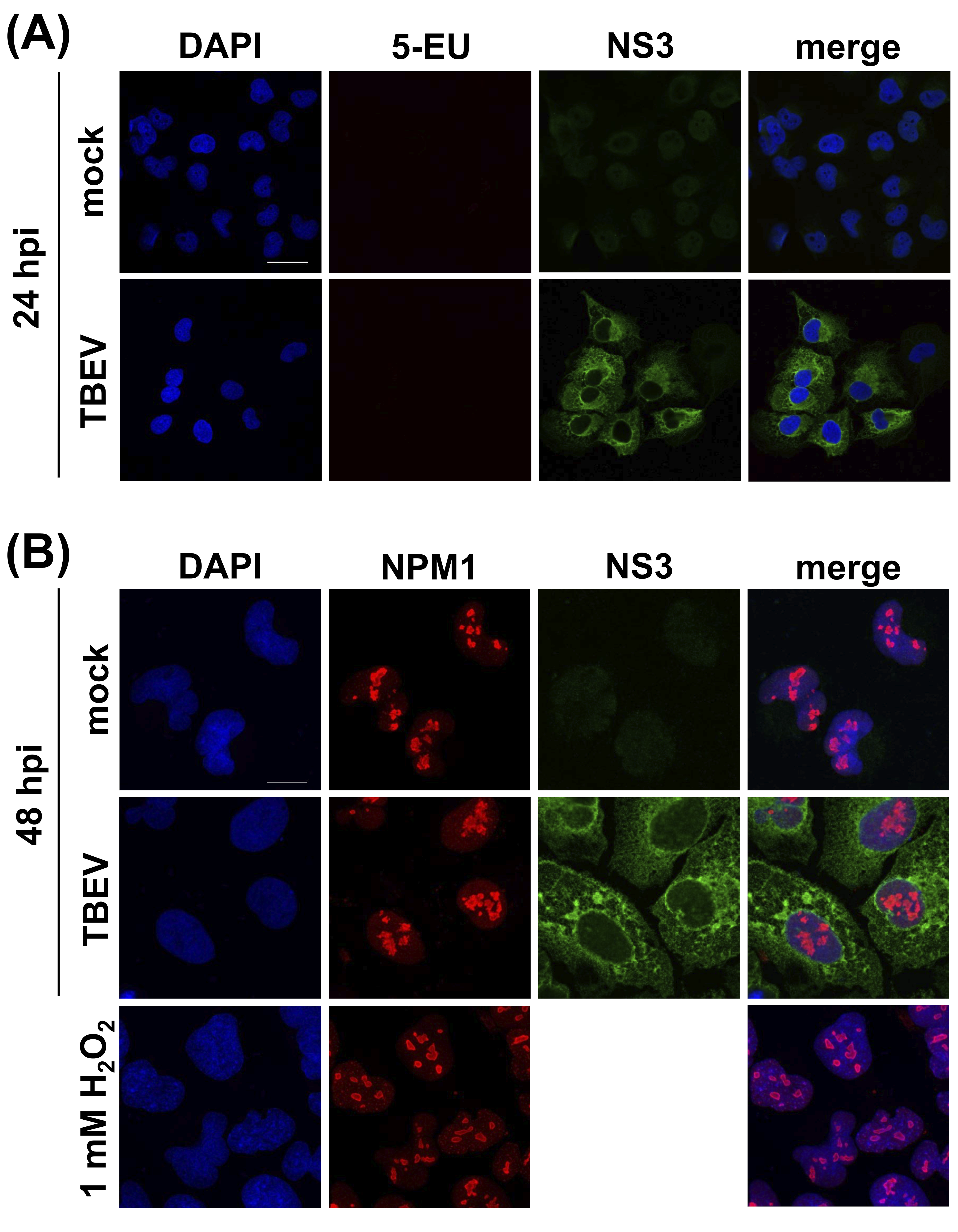

Supplement: S5 Fig — (A) DAOY cells were infected with TBEV Hypr strain (MOI 5) and at indicated time intervals incubated for 2 hours with EU-free cultivation medium. Fixed cells underwent the Click reaction using 10 μM biotin picolyl azide followed by fluorescent labelling with streptavidin-DyLight549. Cells were co-stained with anti-NS3 antibodies; signal was further visualized using anti-chicken DyLight488 antibodies. Scale bar represents 200 μm. (B) DAOY cells were either infected with TBEV Hypr strain (MOI 5) and fixed at 48 hours p.i. or treated with 1 mM hydrogen peroxide for 45 minutes before the fixation. Anti-NPM1 antibodies with the secondary DyLight594-conjugated antibodies were used for the visualization of nucleoli. Scale bar represents 80 μm. (TIF) [file pntd.0007745.s005.tif]

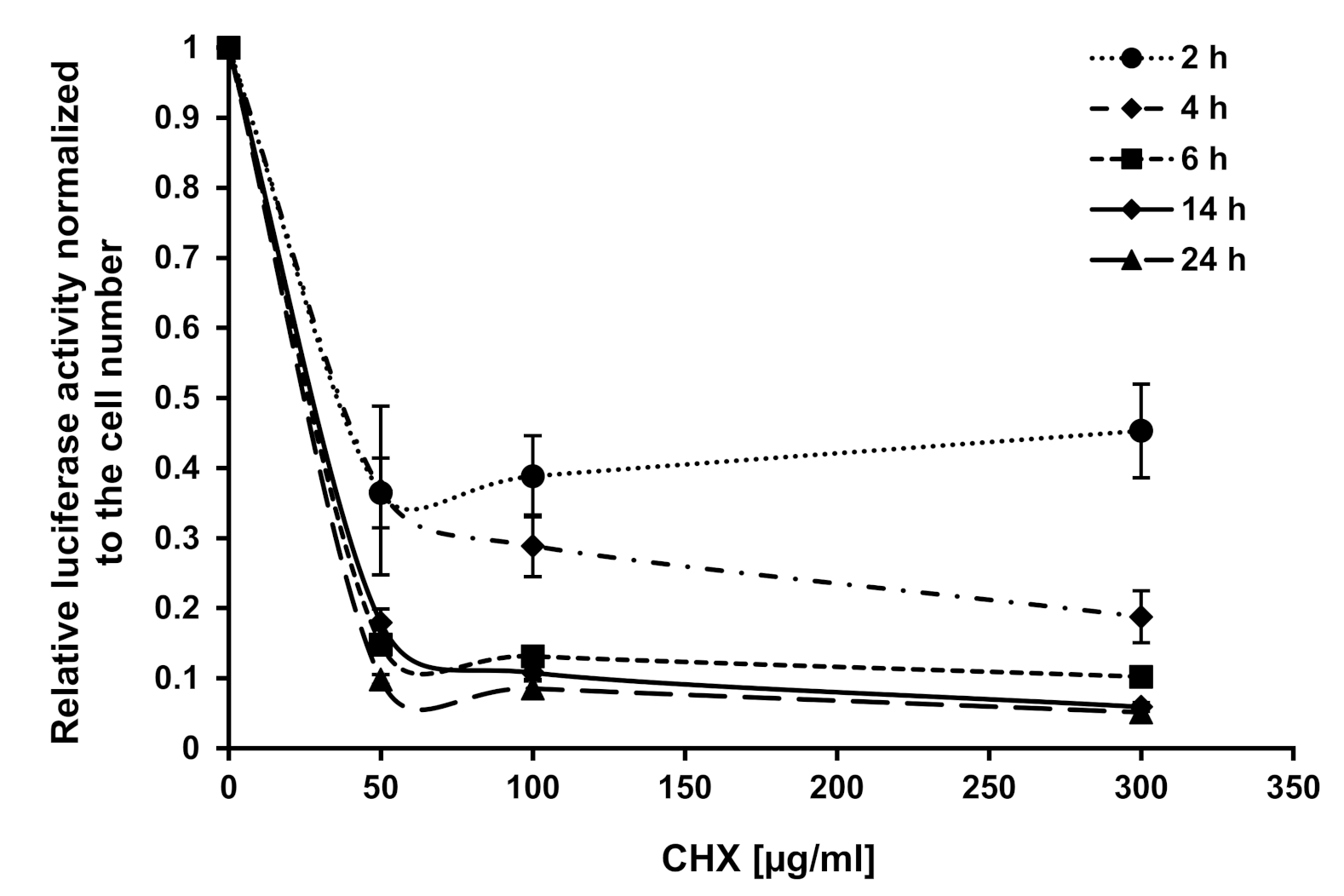

Supplement: S6 Fig — DAOY cells were transfected with 100 ng of pRL-CMV reporter vector expressing RL and subsequently treated with CHX (50, 100 or 300 μg/ml) for time periods indicated. At 24 hours p.t. cell viability as well as luciferase activity was analysed. Data are representative of two independent experiments and values are expressed as mean with SEM. (TIF) [file pntd.0007745.s006.tif]
