# Supplementary material for: Tick-borne encephalitis virus inhibits rRNA synthesis and host protein production in human cells of neural origin
Source: PLoS Negl Trop Dis. 2019 Sep 27;13(9):e0007745. doi: 10.1371/journal.pntd.0007745 (PMC6785130; doi:10.1371/journal.pntd.0007745)
Supplement: S1 Table — (PDF) [file pntd.0007745.s007.pdf]

S1 Table: List of used primers.

Primer	Source	Identificator
RSAD2-F<br>CAAGACCGGGGAGAATACCTG	Primer bank (https://pga.mgh.harvard.edu/primerbank/)	19923667a3
RSAD2-R<br>GCGAGAATGTCCAAATACTCACC	Primer bank (https://pga.mgh.harvard.edu/primerbank/)	19923667a3
mGFP-F<br>CCCCTGCCCTTCGCTTATGA	this paper	N/A
mGFP-R<br>TGCTTCGCTCCCACGAGTAG	this paper	N/A
5S rRNA-F<br>GTCTACGGCCATACCACCC	this paper	N/A
5S rRNA-R<br>AAAGCCTACAGCACCCGG	this paper	N/A
HPRT-F<br>TGACACTGGCAAAACAATGCA	Vandesompele et al., 2002	N/A
HPRT-R<br>GGTCCTTTTCACCAGCAAGCT	Vandesompele et al., 2002	N/A
GAPDH-F<br>CCACATCGCTCAGACACCAT	Teng et al., 2012	N/A
GAPDH-R<br>GGCAACAATATCCACTTTACCAGAGT	Teng et al., 2012	N/A
POLR1A-F<br>GCGATCCGTTGTCCGAAAG	this paper	N/A
POLR1A-R<br>GGTGGGTGTTAAGTATCCTCGTT	this paper	N/A
TBEV E01-F<br>GCAGCCAGATGCCCAACAATGG	this paper	N/A
TBEV E01-R<br>TCTTTTTTGCCTCACAAGCCGCCT	this paper	N/A

Supplementary references

Teng TS, Foo SS, Simamarta D, Lum FM, Teo TH, Lulla A, et al. Viperin restricts chikungunya virus replication and pathology. The Journal of clinical investigation. 2012;122(12):4447-60.

Vandesompele J, De Preter K, Pattyn F, Poppe B, Van Roy N, De Paepe A, et al. Accurate normalization of real-time quantitative RT-PCR data by geometric averaging of multiple internal control genes. Genome biology. 2002;3(7):Research0034.
